# Selective toxicity of antibacterial agents—still a valid concept or do we miss chances and ignore risks?

**DOI:** 10.1007/s15010-020-01536-y

**Published:** 2020-12-23

**Authors:** Axel Dalhoff

**Affiliations:** Christian-Albrechts-University of Kiel, Institue for Infection Medicine, Brunswiker Str. 4, D-24105 Kiel, Germany

**Keywords:** Selective toxicity, Eukaryotic targets, Mitochondria, Metallo-matrix proteinases, Read through, DNA damage repair, Helicases, Neuroprotection

## Abstract

**Background:**

Selective toxicity antibacteribiotics is considered to be due to interactions with targets either being unique to bacteria or being characterized by a dichotomy between pro- and eukaryotic pathways with high affinities of agents to bacterial- rather than eukaryotic targets. However, the theory of selective toxicity oversimplifies the complex modes of action of antibiotics in pro- and eukaryotes.

**Methods and objective:**

This review summarizes data describing multiple modes of action of antibiotics in eukaryotes.

**Results:**

Aminoglycosides, macrolides, oxazolidinones, chloramphenicol, clindamycin, tetracyclines, glycylcyclines, fluoroquinolones, rifampicin, bedaquillin, ß-lactams inhibited mitochondrial translation either due to binding to mitosomes, inhibition of mitochondrial RNA-polymerase-, topoisomerase 2ß-, ATP-synthesis, transporter activities. Oxazolidinones, tetracyclines, vancomycin, ß-lactams, bacitracin, isoniazid, nitroxoline inhibited matrix-metalloproteinases (MMP) due to chelation with zinc and calcium, whereas fluoroquinols fluoroquinolones and chloramphenicol chelated with these cations, too, but increased MMP activities. MMP-inhibition supported clinical efficacies of ß-lactams and daptomycin in skin-infections, and of macrolides, tetracyclines in respiratory-diseases. Chelation may have contributed to neuroprotection by ß-lactams and fluoroquinolones. Aminoglycosides, macrolides, chloramphenicol, oxazolidins oxazolidinones, tetracyclines caused read-through of premature stop codons. Several additional targets for antibiotics in human cells have been identified like interaction of fluoroquinolones with DNA damage repair in eukaryotes, or inhibition of mucin overproduction by oxazolidinones.

**Conclusion:**

The effects of antibiotics on eukaryotes are due to identical mechanisms as their antibacterial activities because of structural and functional homologies of pro- and eukaryotic targets, so that the effects of antibiotics on mammals are integral parts of their overall mechanisms of action.

**Electronic supplementary material:**

The online version of this article (10.1007/s15010-020-01536-y) contains supplementary material, which is available to authorized users.

## Introduction

The theory of selective toxicity was established by Paul Ehrlich. He defined antibacterial agents as “substances with an exclusive affinity for bacteria acting deleteriously or lethally on these alone, while at the same time, they possess no affinity for the normal constituents of the body…” [1–3]. The selective toxicity of ß-lactams, for example, is considered to be due to their affinity to penicillin binding proteins (PBPs) and inhibition of biosynthesis of bacterial cell walls, both being unique to prokaryotes [4, 5]. Sulfonamides are antimetabolites of para-aminobenzoic acid (PABA), so that they compete with folic acid synthesis in bacteria, whereas eukaryotes lack this system [6, 7]. Thus, antibacterial agents interact specifically with targets either being unique to bacteria or being characterized by a dichotomy between pro- and eukaryotic pathways with high affinities of agents to the bacterial- rather than eukaryotic target.

However, it is well documented that aminoglycosides [8, 9], fluoroquinolones [10], tetracyclines [11, 12], macrolides [13, 14], and even ß-lactams [15, 16], optimized for treatment of bacterial infections exert antiviral-, antifungal-, antiparasitic-, and/or antineoplastic effects. It is difficult to comprehend that a monocausal activity relationship allows enough flexibility for such polypharmacological effects. It has been demonstrated that e.g. aminoglycosides [17–19] and tetracyclines [20] bind to a variety of targets. Such alternative binding sites may help to explain the pleiotropic actions of antibacterial agents against pro- and/or eukaryotic organisms.

The interaction of antibacterial agents (antibiotics in the following) with mammalian targets is known since long. The discovery of hypoglycaemic sulfonamides can be attributed to the observation in 1942 that treatment of typhoid fever with the sulphonamide 2254RP caused hypoglycaemia in some patients [21]. A number of sulfonamides are used in the treatment of protozoal infections in animals and humans. In addition, immunomodulatory activities in particular of macrolides [22, 23], fluoroquinolones [24, 25], and almost every other drug class [26, 27] demonstrate that antibiotics affect eukaryotic cells, too, probably due to a physicochemical interaction with membranes thus triggering intracellular signaling cascades in pro- and eukaryotes [28], so that interspecies and interkingdom communication is affected [29–32]. These examples indicate the paradigm of selective toxicity is oversimplified. Instead, agents interfere with multiple targets in pro- as well as eukaryotic cells due to compound- and target promiscuity [33–35] and target homology as will be demonstrated below.

This review summarizes data describing multiple modes of action of antibiotics and is limited exclusively to a synopsis of studies describing interactions of antibiotics with pro- and eukaryoric targets although the clinical condition may be complex and multifactorial. Thus, clinical improvement may result from pleiotropic actions of an antibiotic which, however, should not be mentioned as this review is focussed on the targets. Only those agents are mentioned which are used clinically for treatment of bacterial infections; their structural derivatives optimized for non-antiinfective activities will not be discussed. Phenotypic descriptions of drug effects will not be discussed either. Comprehensive reviews have aggregated a plethora of data describing the phenotypes of action of polyfunctional antibiotics [36–42]. The reader is kindly referred to these summaries for further information. Adverse drug reactions, drug/drug interactions, and indirect effects mediated by oxidant scavenging activities, immunomodulation, and beneficial- or detrimental effects resulting from microbiome imbalance, etc. will not be discussed either. To emphasize a priori, most of the therapeutic opportunities mentioned below have not been reviewed by regulatory authorities and the preclinical and/or clinical evidence supporting these potential novel indications varies widely. The majority of preclinical studies were planned as hypothesis generating experiments and most clinical studies have been designed as retrospective observational rather than prospective, randomized clinical studies. But still these data suggest that therapeutic options of an antibiotic treatment may likely be more diverse than expected.

## Non-antimicrobial activities of antibiotics beyond structural and mechanistic boundaries

Detailed information about non-antimicrobial actions of antibiotics is provided in the supplemental material. Data compiled in Tables S1 and S2 confirm that antibiotics of almost every drug class interact with cellular- and in particular mitochondrial functions like autophagy and apoptosis/caspase mediated proteolysis as well as activities of matrix metalloproteinases (MMPs) thus exerting pleiotropic effects including anti-neoplastic activities. However, review and analysis of published data is difficult, since four variables had a significant impact on the data generated. First, the use of different methods results in divergent outcomes [43]. Second, antibiotics affected cellular functions also indirectly via interaction with cytokines and/or signaling cascades. Such systems are frequently redundant and pleomorph. Many cytokines are synthesized by more than one cell and different cells may secrete different cytokines with dissimilar activities and different stimuli may trigger diverse effects [24, 25]. Consequently, indirect effects of antibiotics on cellular functions via immunomodulation are not uniform but differ according to cell lines used and targets studied. Three examples should be mentioned pars pro toto: azithromycin and moxifloxacin decreased respiratory epithelial cell derived MMP1 and -3 concentrations, whereas both agents increased MMP secretion from MRC-5 fibroblasts [44]. Concentrations of doxycycline to inhibit MMP-9 synthesis to 50% (IC_50_) ranged in different cell lines from 1 to 608 μM [43, 45]. IC_50_-values of azithromycin for inhibition of proliferation and induction of apoptosis in HeLa-, cervical- and gastric cancer cells were 15.66, 26.05 and 91.00 mg/L [46]. Third, the parameter measured is not necessarily congruent to the mode of action of an antibiotic. Rifampicin and para-aminosalicylic acid, for example, inhibited secretion of MMPs secondary to an inhibition of prostaglandin synthesis [47, 48], so that these antibiotics targeted the endocrine system but not MMP synthesis. Fourth, antibiotic concentrations used in in vitro experiments scatter over a broad range and were frequently unphysiologically high. However, humanized doses were administered to experimental animals and standard doses were used in clinical studies on e.g. anti-neoplastic effects of antibiotics – even in monotherapy—or inhibition of MMP activities. For example, pancreatic cancer cell lines were exposed to 400 mg/L each of ciprofloxacin, moxifloxacin, and gatifloxacin; their maximal serum concentrations following oral standard doses are as low as 2.3-, 5.0-, and 3.8 mg/L [49, 50]. Patients with rosacea or periodontitis are treated with subantimicrobial doses of 40 and 20 mg b.i.d. doxycycline. This apparent discrepancy between pre-clinical and clinical study data and authority-approved indications remains unexplained. The more important it is to scrutinize the relevance of preclinical findings in proof of principle studies at the earliest possible time using alternative strategies [51, 52].

### Antibiotics target mitochondrial functions and inhibit cancer cell growth

Mitochondrial metabolism is essential for e.g. energy conversion and regulation of membrane potential. Mammalian mitochondrial functions represent targets for antibiotics as documented already > 50 years ago [53–69]. Antibiotics interact not only with prokaryotic- and mitochondrial ribosomes but with eukaryotic 80S-ribosomes as well, so that human mitochondria and 80S-ribosomes represent a cancer target for antibiotics, too [70–74]. Data summarized in Table S1 demonstrate that cancer cell growth was affected by aminoglycosides, macrolides, tetracyclines, oxazolidinones, chloramphenicol, clindamycin, rifampicin targeting mitochondrial translation, quinolones targeting mitochondrial topoisomerases, and bedaquilin targeting mitochondrial ATP-synthesis [75]. However, the mitochondrial membrane may constitute a permeation barrier thus explaining that oxazolidinones, chloramphenicol and tetracyclines were significant inhibitors, while macrolides, clindamycin and aminoglycosides were poor inhibitors of mitochondrial protein synthesis [68]. Beta-lactams affected mitochondrial functions due to inhibition of carnitine/acylcarnitine transporter (Table S1). In addition, the agents also affected signaling cascades, micro-RNAs, and acted as immunomodulators or anti-inflammatory agents, so that antineoplastic activities of antibiotics may be due to interactions with multiple targets. Clinical trials indicated that aminoglycosides, macrolides, tetracyclines and fluoroquinolones, clarithromycin, and ciprofloxacin in particular, reduced mortalities in cancer patients, even as mono-therapeutics [76–82]. Synergistic effects were recorded in combination with anti-neoplastic agents. Thus, it may be plausible to prefer these agents to other antibiotics in cancer patients to benefit from their anti-neoplastic activities and their synergistic effects with other cancer treatment modalities. Antibiotics could be used not only for prophylaxis- or treatment of bacterial infections, but also for adjuvant therapy in cancer patients.

### Antibiotics inhibit metallo-matrix-proteinases

MMPs are Ca^2+^ containing endopeptidases with an essential Zn^2+^ bound to three histidine residues in the conserved catalytic region. MMPs are characterized by a broad spectrum of substrate specificities and are essential for migration, invasion and degeneration of cells. MMPs are involved in e.g. bone remodelling, angiogenesis, and wound healing, etc., but also in arthritic destruction, pulmonary fibrosis, and cancer development, etc. [83–85]. MMP activity is regulated amongst others by tissue inhibitors of metalloproteins (TIMPs), which chelate the catalytic zinc atom thus inactivating MMPs [86]. Therefore, antibiotics known to chelate with bi- and trivalent cations like tetracyclines [87–89] and fluoroquinolones [90] should theoretically inactivate MMPs. The oxazolidin ring of linezolid and its peptide bond may chelate with zinc/calcium. Furthermore, vancomycin [91], bacitracin [92, 93], isoniazid [89], macrolides [94–96], ß-lactams [97–107], nitroxoline [108, 109], and chloramphenicol [110] formed chelates or complexes with zinc and calcium, whereas streptomycin did not [89].

Data summarized in Table S2 show that expectedly all these agents inhibited MMPs except fluoroquinolones and chloramphenicol, which unexpectedly induced MMPs. It is difficult to distinguish whether the described effects are due to inhibition of enzyme activities or inhibition of MMP synthesis. Few investigators only have analysed enzyme activities or gene expression, whereas MMP concentrations in supernatants of cell cultures have been analysed in most of the studies. A decrease in MMP concentrations as a consequence of antibiotic treatment could be demonstrated in patients with orthopaedic infections treated with ß-lactams [111, 112] and daptomycin [113], as well as pulmonary diseases treated with clarithromycin and azithromycin [114, 115]; doxycycline reduced MMP concentrations in patients with CF, COPD, and tuberculosis [116–119], chronic wounds [120–128], abdominal aortic aneurysm [129–132], ophthalmological diseases [133, 134], and in patients with acute lung injury following cardiac bypass [135]. Marketing authorizations have been granted for treatment of rosacea and periodontal diseases with sub-antimicrobial doses of doxycycline due to MMP inhibition [136–142]. Interestingly, several disinfectants inhibiting MMPs via cation-chelation are used clinically in dentistry [143–152]. Aminoglycosides exhibited anti-neoplastic effects as well, which were due to drug-induced increases in read-through of premature stop codons and not, as expected, inhibition of MMPs due to a lack of chelation with zinc (Table S2).

## Drug class specific non-antimicrobial activities of antibiotics

### Sulfonamides

Although sulfonamides are antimetabolites of PABA [6, 7], they are multifunctional drugs as their -SO2NH- (or -OSO2NH-, -NHSO2NH-) moieties interact with metal ions, amino acid residues, as well as DNA- or RNA-moieties. The antibacterial sulfonamide 2254RP exerted hypoglycaemic activities [6, 7, 21]. Sulfanilamide (Prontosil^®^) inhibited human carbonic acid anhydrases playing crucial physiological roles including pH regulation, gluconeogenesis, respiration, etc. due to the interaction of its thiol group with the Zn^2+^ ion in the active centre of the enzyme. Other sulfonamide-derivatives exhibited anti-obesity-, diuretic-, antithyroid-, anti-tumor-, anti-neuropathic pain-, anti-inflammatory activities, and acted as serotonin antagonists and protease inhibitors, too [153, 154]. However, non-antibacterial sulfonamides are almost inactive against bacteria due to the fact that the best position of the aniline group and the acidic sulfonamide moiety to mimic PABA is the para-substitution about the benzene ring. This positioning is essential for antibacterial activities of sulfonamides [155].

### ß-lactams

Beta-lactam antibiotics mimic the structure of the acyl-d-Ala-d-Ala C peptide chain terminus of the growing cell wall and react with PBPs to form an acyl-enzyme complex [4, 5]. The D-Ala-D-Ala building block as well as the structure of the bacterial cell wall is considered to be unique to prokaryotes. Consequently, PBPs and class A, C, and D ß-lactamases being acyl serine transferases are considered to be bacteria specific [156]. But structural homologies between bacterial- and human serine proteases are conceivable as eukaryotic acyl serine transferases like trypsin, thrombin, etc. transfer the electrophilic group of e.g. peptide carbonyl donors to an acceptor. The carbonyl donor is transpeptidated with the acceptor when the acceptor carries an aminogroup – as happens in the course of cell wall biosynthesis. Most of the approximately 1,000 bacterial serine proteases like the D-Ala-D-Ala carboxypeptidases are single domain proteases in prokaryotes with functional specialisation [157], while e.g. trypsin-, subtilisin-, and Lon-protease families account for multi-domain proteases with inter-kingdom distribution [157–159]. The structures of the catalytic domains, however, are highly conserved, so that ß-lactams may interact with mammalian serine proteases, too.

Cefoperazone prevented α_1_-antitrypsin inactivation [160] and ceftazidime inhibited neutrophil elastase activity [161]. Additional reports are scarce although non-antibacterial lactams interacted with many mammalian targets [15, 16, 162–165]. Whether this can be attributed to the marginal activities of commercially available ß-lactams, or if they have not been exploited systematically is uncertain. Vice versa, it has been shown that inhibitors of eukaryotic serine proteases increase activities of ß-lactams in susceptible and even resistant bacteria just because of structural homologies between PBPs and human serine proteases.

The PBP- and ß-lactamase core protein fold is highly conserved and is present in human enzymes, too [166–168]. These human metallo-ß-lactamases (hMBLs) catalyse pleiotropic reactions on which various catalytic, regulatory and structural activities are based [169–173]. One  of the ≥ 18 known hMBL is the intra-mitochondrial membrane organizing serine ß-lactamase like protein (LACTB) which binds two metal ions and acts as a tumor suppressor that modulates lipid metabolism and cell state [174–177]. Other hMBLs inactivate not only anti-neoplastic agents but also penicillin G but are inhibited by sulbactam and clavulanic acid [178] as well as 7-aminocephalosporinic acid, cephalosporin C, cefotaxime, and ceftriaxone, while compounds with a penicillin-, carbapenem- or monobactam-core were inactive [179]. Although the cephalosporins and ß-lactamase inhibitors tested were active against purified hMBLs only but exhibited no activities in cell based assays these data could provide a basis for the development of selective hMBL-inhibitors thus inhibiting inactivation of anti-neoplasic agents ot interacting with tumorigenesis. Furthermore, ß-lactams inhibited tumor growth due to inhibition of mitochondrial carnitine/acylcarnitine transporter (Table S1). In addition, sequence homologies between PBPs and DNA-polymerase-α have been described, so that ß-lactams affect proliferating eukaryotic cells in the S-phase [180–185]. Cephalosporins inhibited growth of various cell lines by three- to 25-fold lower concentrations than penicillins, whereas clavulanate, sulbactam, and monobactams were inactive. If the anti-neoplastic effect of some ß-lactams summarized in Table S1 may or may not be linked to inhibition of DNA-polymerase-α and/or inhibition of mitochondrial functions remains an open question.

Beta-lactams, ceftriaxone in particular, act as neuroprotectants in various models of neurological and neurodegenerative diseases, such as Alzheimer's disease, Parkinson, epilepsy, strokes, etc. which have been linked to dysfunction of excitatory amino acid transporters (EAATs). EAATs exhibit structural homologies between eukaryotic, bacterial and archaeal glutamate transporters [186–201]. The activities of ß-lactams were due to an increased expression of glutamate transporter GLT1 (EAAT2) [202–206]. However, the mechanisms by which ß-lactams enhance gene expression uniquely of glutamate transporter EAAT2 and not other glutamate transporters remains unanswered. Hypothetically, ß-lactams either enhance EAAT2 gene expression through NF-κB mediated modulation of EAAT2 promotor activity, and/or act as chelators. Binding of NF-κB to one out of four NF-κB binding sites of the EAAT2 promotor was increased by ceftriaxone thus activating transcription. Ceftriaxone activated also other NF-κB signalling pathways being indirectly involved in ceftriaxone mediated EAAT2 promotor activation [207, 208]. Alternatively or in parallel ß-lactams may chelate with copper, an essential trace element. Ceftriaxone bound copper effectively, so that it could ameliorate neurodegenerative diseases [103, 106]. In addition, ß-lactams modulate cytokine activity and regulate gene expression in mammals through a covalent binding to cytokines or proteins [209–214]. Not only ß-lactams but minocycline, rapamycin inhibiting a serine/threonine protein kinase, and rifampicin inhibiting bacterial RNA polymerase acted as neuroprotectants [215, 216]. These findings indicate that the mechanism(s) of neuroprotective functions of such diverse classes of antibiotics are far from being understood.

### Aminoglycosides

Aminoglycosides inhibit bacterial protein synthesis by binding to the A-site on the 16S ribosomal RNA of the 30S ribosome thus inducing codon misreading resulting in mistranslation. In addition, aminoglycosides interact with a great variety of RNAs. They bind to hammerhead ribozyme, tRNA-Phe, the Rev response element transcriptional activation region in human immunodeficiency virus, the ribozyme from hepatitis delta virus, group I self-splicing introns, etc. because of their polycationic nature [17–19]. Contrary to the previous assumption, aminoglycosides interact with eukaryotic ribosomes, too. Analysis of crystal structures of 80S ribosomes in complex with aminoglycosides revealed that aminoglycosides bind to multiple sites on both subunits, so that multiple modes of action on the translation mechanism are probable [217–219]. Translation, elongation and termination are altered in a manner that induced read-through of premature stop codons (PTCs) (220–222, and Table S1). These findings have led to their consideration as potent drugs to treat human diseases caused by PTCs [221]. Although the modes of action of aminoglycosides on eukaryotic ribosomes and their potential clinical efficacy in treatment of non-infectious diseases has been elucidated quite recently [220–222], their efficacies in treatment of genetic disorders has been described phenotypically already in 1985, so that many data could be accumulated. Comprehensive recent reviews demonstrate that aminoglycoside-induced mutation suppression has not only been confirmed in a variety of preclinical in vitro and in vivo models but translates also into the clinical arena [223–226] (Table 1). Gentamicin, the investigational agents geneticin (G-418) and G-418 derivatives were the most frequently studied aminoglycosides; tobramycin, amikacin, paromomycin, neomycin, sisomycin, kanamycin, lividomycin, and hygromycinB were evaluated as well. While the major gentamicin compnts lack read-through activity the minor compnts, B1, X1, and G-418 caused read-through of PTCs [227, 228]. Aminoglycosides were initially used clinically as read-through agents in particular in the treatment of cystic fibrosis patients but concerns over the safety profile of aminoglycosides led to a search for alternatives.

**Table 1:** Translational read-through studies

Agents	Disease	Study design	Major findings	Ref’s
Aminoglycosides				
GEN, TOB, AMK, NEO, KAN, SIS, LIV, PAR, HYGB, geneticin	Patients with cystic fibrosis, DMD, ataxia telangiectasis, hemophilia	In vitro cellular, ex vivo patient derived cells, in vivo disease models, clinical observations	Variable in vitro and in vivo findings. Read-through has been confirmed in patients suffering from cystic fibrosis, DMD, ataxia telangiectasis, and patients with hemophilia A and –B Treatment duration up to several months	[225–228]
Macrolides				
ERY, OLE, SPIR, TYL	n.a	In vitro molecular, *lacZ* ± UAG or UGA	Substantial levels of read-through; erythromycin was most effective	[244]
AZM, TYL	n.a	In vitro molecular, 80S ribosome,	Little read-through activity	[250]
ERY, AZM	Adenocarcinoma	Ex vivo clinical specimen and transgenic mouse model	Azithromycin induced read-through at a 100-fold lower concentration than erythromycin	[251, 252]
TYL	Colorectal cancer	Ex vivo clinical specimen and transgenic transgenic mouse model	Restoration of APC gene function	[253]
ERY	Adenomatous polyopsis	pilot study in nine patients	In the majority of patients the treatment led to a decrease in cumulative adenoma burden, median reduction in cumulative adenoma size and median reduction in adenoma number	[254, 255]
Phenicols				
Chloramphenicol	n.a	In vitro molecular*, lacZ* ± UAG or UGA	Extensive levels of read-through	[244, 273]
Chloramphenicol	Breast cancer	In vitro cellular, murine breast cancer EMT6 cells. The genetic code of the amino acid selenocysteine is UGA	Most selenoproteins are oxidoreductases involved in housekeeping processes and stressresponse. Increase of stop codon read through resulting in high levels of mistranslation of the UGA-code in mammalian cells, so that the cellular redox control is dysregulated	[274]
Chloramphenicol	Mucopoly-saccharidosis	Ex vivo clinical specimen. Fibroblasts from patients with nonsense mutations or stop codons leading to null or low enzymic activity	Nonsense mutations are present in 2/3 of the patients, stop codon read through is a potential alternative to achieve enhanced enzyme activity. Chloramphenicol increased in enzyme activity 100-fold. However, cDNA sequencing showed that only the alleles without the nonsense mutation were being amplified, possibly due to the targeting of nonsense alleles to nonsense-mediated mRNA decay. Chloramphenicol may act through a mechanism other than stop codon read-through	[275]
Chloramphenicol	n.a	In vitro molecular. Read-through level of *cspC* UGA in *E. coli*	Chloramphenicol increased read-through	[276]
Oxazolidinones				
Linezolid	n.a	In vitro molecular*, lacZ* ± UAG or UGA	Induction of read-through	[273]
Linezolid, Rχ-01	n.a	In vitro molecular*, lacZ* ± UAG or UGA	Linezolid induced read-through 5-times less efficient than the investigational oxazolidin Rχ-01	[277]
Tetracyclines				
Tetracycline	n.a	In vitro molecular. Read-through level of *cspC* UGA in *E. coli*	Tetracycline increased read-through	[275]
Doxycycline	Breast cancer	In vitro cellular, murine breast cancer EMT6 cells. The genetic code of the amino acid selenocysteine is UGA	Most selenoproteins are oxidoreductases involved in housekeeping processes and stressresponse. Increase of stop codon read through resulting in high levels of mistranslation of the UGA-code in mammalian cells, so that the cellular redox control is dysregulated	[274]

*n.a.* not applicable; *GEN* gentamicin; *TOB* tobramycin; *AMK* amikacin; *NEO* neomycin; *KAN* kanamycin; *SIS* sisomycin; *LIV* lividomycin, *PAR* paromomycin, *HYGB* hygromycinB; *AZM* azithromycin; *ERY* erythromycin; *OLE* oleandomycin; *SPIR* spiramycin; *TYL* tylosin; *DMD* Duchenne muscular dystrophy; *lacZ* reporter gene ± UAG or UGA stop codon = ß-galactosidase expression

Several specific features possibly limit the use of aminoglycosides in the treatment of genetic disorders: First, the identity of the stop codon and second, the sequence context surrounding it have an impact on the aminoglycoside mediated readthrough [222, 229–233], so that aminoglycosides can not suppress PTCs equally well in all patients, thus probably necessitating individualized diagnostic procedures. Third, mitochondrial dysfunction may occur in patients with specific polymorphisms in their 12S rRNA [234]. Fourth, aminoglycosides impair cell respiration leading to a superoxide overproduction. Furthermore, oxidative damage of mitochondrial aconitase leads to an accumulation of free ferrous iron in mitochondria, so that ultimately cells undergo apoptosis via the Fenton reaction (235).

### Macrolides

Macrolides inhibit protein synthesis by binding to the bacterial ribosome in the region of the nascent peptide exit tunnel. Binding of a macrolide in the exit tunnel obstructs the passage of the nascent peptides, so that overall protein synthesis rapidly declines and peptidyl-tRNAs are accumulated. Therefore, it was previously suggested that macrolides inhibit the production of cellular polypeptides completely by preventing the exit of nascent peptide chains. Recent findings revealed that macrolides, instead of directly binding to the peptidyl transferase centre and disrupting its structure, bind in the exit tunnel one or more nanometres from the peptidyl transferase centre, so that the diameter of the exit tunnel is decreased. This results in steric clashes with nascent peptides leading to a stalling of ribosome function. Despite the narrowing of the exit tunnel sufficient room is still left, so that some proteins may bypass the narrowed exit tunnel and become partially or fully synthesized, while other proteins cannot bypass the obstruction. Inhibition of protein synthesis by macrolides depends on the properties of the polypeptide being synthesized by the drug-bound ribosome. The ability to bypass is not dependent on the entire sequence of the synthesized protein, but rather on its N-terminal sequence. In addition/or other mechanisms may cause differential impacts of macrolides on protein synthesis. Perturbed conformation of rRNAs in the P-site, which can lead to a decrease in translation rate, or reorientation of rRNAs might affect the stereochemistry of the A-site thereby preventing peptide bond formation [236–241]. Macrolides stall not only the ribosome within specific sequence contexts, but also induce translation errors [238, 242]. This effect is probably due to an inhibition of peptide bond formation and/or peptide release. This event could in turn alter kinetics of translation resulting in an increased chance of read-through. All the 14-membered and 16-membered lactone ring macrolides caused an extensive stop codon read-through of PTCs in the in vitro system studied [242] (Table 1). In addition, macrolides can also stimulate ribosomal frameshifting, which, however, is linked to resistance mechanisms [233, 237] and thus not relevant in the context of this review.

Read-through activities of macrolides were recorded in eukaryotes although only bacterial- or mitochondrial ribosomes are susceptible to macrolides, whereas ribosomes isolated from archaea or eukaryotic cytoplasmic ribosomes are resistant [243–246]. If read-through in eukaryotes is due to an interference with mitochondrial functions only has not been addressed. Nevertheless, a clinical study investigating the efficacy of erythromycin treatment for read-through of APC gene stop codon mutations in familial adenomatous polyposis has been initiated [247] and the impact of macrolides on stop codon read-through has been proven in pro- and eukaryotic species as well as patients (Table 1) [242, 248–253].

Furthermore, macrolides are prokinetic agents due to their motilin receptor stimulating activities. Fourteen- and 15- but not 16 membered macrolides act as motilin receptor agonists. Erythromycin affects two different pathways; at a low dose (40 mg) it activates an intrinsic cholinergic pathway, whereas higher doses (200–350 mg) act on motilin receptors on enteric nerves and smooth muscle [254–258]. Erythromycin itself exerts almost no prokinetic activity but its antibacterially inactive degradation intermediate 8,9-anhydro-6,9-hemiketal serves as a motilin receptor agonist and is then further metabolized into erythromycin-6,9;9,12-spiroketal [259]. The disadvantages of the use of macrolides as prokinetic agents are that motilin-induced contractions induce hunger feelings through a cholinergic pathway [256] and that macrolide-resistance might develop.

Another target for macrolides in eukaryotes has been described recently. Roxithromycin inhibited the cellular differentiation of the rice blast fungus *Magnaporthe oryzae* [260]. The gene *mocdc27* (*M. oryzae* Cell Division Cycle 27) encoding appressorium formation is involved in growth inhibition of the fungus by roxithromycin. However, *mocdc27* knock down mutants formed appressoria identical to those of the wild-type fungi. Therefore, it may also be likely that a complex of roxithromycin-MoCDC27 affects another molecule involved in appressorium formation. *mocdc27* encodes a protein that is highly homologous to the anaphase promoting complex/cyclosome(APC/C) subunit (CDC27/APC3) of other eukaryotes. CDC27 homologs have been detected in yeasts, plants, and mammals. CDC27 plays a key role in colorectal cancer as CDC27 expression is significantly correlated with tumor progression and poor patient survival [261]. These findings and the interaction of macrolides with MoCDC27 may help to explain why macrolides exert anti-neoplastic effects (Table S1).

### Chloramphenicol

Chloramphenicol inhibits translation by binding with its nitrobenzyl ring to several nucleotides of the 23S rRNA at the A site of the peptidyl transferase centre. The aromatic ring of the ribosome-bound chloramphenicol overlaps with the A-site, so that the aminoacyl moiety of incoming aminoacyl-tRNA cannot properly attach to peptidyl transferase centre. Originally, inhibition of translation was thought to be universal. However, the inhibitory activity of chloramphenicol is context specific. Inhibition depends on the nature of specific amino acids in the nascent chain and the identity of the residue entering the A site [262–265]. Chloramphenicol readily binds to mitochondrial- but not to mammalian cytoplasmic ribosomes [266–270]. Data summarized in Table 1 demonstrate that chloramphenicol affected read-through in eukaryotes, too [242, 271–274]. The impact of chloramphenicol on gene expression in eukaryotes is possibly due to alternative mechanisms or downstream effects to inhibition of mitochondrial translation resulting in decreases in cell surface transferrin expression, de novo ferritin synthesis, thus affecting iron dependent respiratory chain activity resulting in an reduced ATP synthesis and eventually in reduced tumor cell growth (Table S1). It has been shown recently that chloramphenicol inhibited appressorium formation in *Magnaporthe oryzae* because of an inhibition of MoDullard, a serine/threonine phosphatase [275]. Alignment and comparison of amino acid sequences of fungal and human origin revealed that five MoDullard orthologs could be identified from the human genome but only caboxy-terminal domain RNA polymerase II polypeptide A small phosphatase 1 (CTDSP1) complemented the MoDullard function and could be inhibited by chloramphenicol. Small serine phosphatases can exhibit multiple functions in a variety of cellular and biological processes. Human CTDSP1 interacts with a variety of proteins like cell division cycle associated protein 3, myelin basic protein, RE1-silencing transcription factor, some of which are involved in cell differentiation [275]. If CTDSP1 may represent a novel target for chloramphenicol in humans has to be verified or falsified. It also has to be examined if interaction of chloramphenicol with CTDSP1 may have an impact on CTDSP1-networks with a variety of proteins being involved in cell differentiation and carcinogenesis. But anyway, an unexpected chloramphenicol target has been identified in humans.

### Oxazolidinones

Most of the studies on the mode of action of oxazolidinones have been performed with linezolid. Linezolid and earlier oxazolidinone derivatives were found to interfere with the early phase of protein synthesis, i.e. the formation of a functional 70S initiation complex with the two ribosomal subunits due to binding of linezolid to both subunits [276–279]. However, biochemical- and target binding studies suggested that oxazolidinones not only interfere with the formation of the initiation complex but interfere with almost each and every step of protein synthesis like elongation factor G dependent translocation during elongation and frameshifting, termination of translation, and nonsense suppression [280]. Also, resistance mutations mapping provided conflicting data. Docking studies and crystallography revealed that linezolid bound to the 50S ribosomal subunit in the A site pocket at the peptidyltransferase centre [280, 281]. Binding to the A site impeded the proper placement of incoming aminoacyl-tRNAs and also binding of other protein synthesis inhibitors like chloramphenicol [280–282]. Linezolid inhibits peptide-bond formation not globally, but rather blocks translation at specific locations within the mRNA in a context-specific manner [283]. Linezolid binds exclusively to mitochondrial ribosomes and leaves cytoplasmic ribosomes unaffected [284]. Linezolid and to a higher degree the investigational oxazolidinone R χ -01, increased frameshifting and stop codon read-through with nonsense suppression [271, 285]. Consequently, linezolid and tedizolid caused mitochondrial dysfunction thus suppressing cancer cell growth and increasing apoptosis [286–289]. Linezolid also inhibited overexpression of mucin MUC5AC in human airway epithelial cells at concentrations of 5 and 20 mg/L due to inhibition of phosphorylation of ERK1/2, which caused the activation of a MAPK pathway member resulting in overexpression of MUC5AC. This effect is analogous to that seen with azithromycin, which reduced mucin production via the NF-κB pathway [290–292].

### Tetracyclines

Tetracyclines inhibit bacterial protein synthesis by preventing the association of aminoacyl-tRNA with the bacterial ribosome. Tetracyclines bind to the 30S ribosomal subunit at high-occupancy tetracycline-binding site (Tet-1) and five other minor binding sites in 16S rRNA. Tetracycline most likely binds complexed with two Mg^2+^ ions at the Tet-1 site near the A-site. A comparison of tigecycline- and tetracycline binding sites to the 70S ribosome and tigecycline binding to the 30S ribosome, respectively, showed that tigecycline was bound only to the Tet-1 site, and secondary binding sites were not observed. Thus, the significance of the other five tetracycline-binding sites is unclear [293, 294]. In addition, tetracyclines bind to various synthetic double-stranded RNAs, suggesting that the double-stranded structures may play a more important role in binding of tetracyclines to RNA than binding to specific base pairs [20]. This also implies that tetracyclines bind to double-stranded RNAs of pro- as well as eukaryotic origin. Furthermore, doxycycline bound to human cytosolic 80S ribosomes, so that human 80S ribosomal translation was modified and the cellular integrated stress response was activated [295, 296]. These findings contribute to an explanation why tetracyclines induced read-through of PTCs [272, 273] and exerted anti-proliferative effects on human cancer cell lines and in patients (Table S1). Favourable clinical outcomes have also been observed with tetracyclines in indications like rheumatoid arthritis, osteoarthritis, Fragile X syndrome, etc. [129, 131, 297–304]. A comprehensive summary of pleiotropic non-antibacterial actions and on-going clinical trials was published in 2010 [11] listing 54 and 88 clinical trials for minocycline and doxycycline, respectively, in indications like e.g. asthma, autism, Parkinson’s disease, amyothrophic lateral sclerosis.

Tetracyclines also exhibit an anti-parasitic effect and affect growth of e.g.

*Plasmodium falciparum, Entamoeba histolytica, Giardia lamblia, Leishmania major, Trichomonas vaginalis,* and *Toxoplasma gondii* [20]. The antiparasitic activity is explained by protein synthesis inhibition in mitochondria. However, *T. vaginalis, G. lamblia,* and *E. histolytica* are devoid of mitochondria but are nevertheless susceptible to tetracyclines. Thus, tetracyclines should affect structures in eukaryotes other than just mitochondria. Five not necessarily mutually exclusive theories are discussed: First, tetracyclines may inhibit translation in bacterial endosymbionts, like Wolbachia in helminths, being essential for parasite survival and reproduction [20, 305, 306]. Second, eukaryotes without mitochondria may contain mitochondria derived organelles like mitosomes and hydrogenosomes [307], so that such organisms contain genes of mitochondrial ancestry. Tetracycline targeted the hydrogenosome of *T. vaginalis* thus causing cell death [308]. *P. falciparum* harbours a plastid (apicoplast) that originated from an eukaryotic algal lineage. This organelle, too, is inhibited by tetracycline [309]. Third, binding of the tetracyclines to double stranded RNA may represent an alternative to the ribosomal target site thus allowing tetracyclines to affect organisms that lack the 16S ribosomal RNA (such as RNA-viruses and some protozoa) [20, 310]. Fourth, tetracyclines are strong chelators of divalent cations other than zinc [311–314], so that tetracyclines interact not only with metallo matrixproteinases (see paragraph 2.2. and Tables S1, S2), but affected also the interaction with double stranded RNA and thus their own uptake into Gram-negative bacteria, and their affinities to pro- or eukaryotic targets [20, 294]. Fifth, tetracyclines acted as pro-apoptotics in various cell-lines [11, 20, 315–319]. Furthermore, tetracyclines are used as valuable tools in biomedical research. The tetracycline-controlled Tet-Off and Tet-On gene expression systems are used to regulate the activity of genes in mammalian cells under various experimental conditions [320–326].

These data imply that tetracyclines could in theory be clinically effective in treatment of a broad variety of non-infectious diseases. However, the question of expanding the indications has not been addressed systematically, so that these findings represent interesting observations but do not yet translate into the clinical arena. Exceptions are marketing authorizations for treatment of rosacea and periodontal diseases with sub-antimicrobial doses of doxycycline.

### Fluoroquinolones

In bacteria fluoroquinolones target both type II topoisomerases, i.e. DNA gyrase and topoisomerase IV [327, 328]. A critical role in the Mg^2+^ dependent interaction between topoisomerases and fluoroquinolones play two key residues in the GyrA and ParC/GrlA subunits of the heterotetrameric structure of topoisomerases. An invariant Ser, or sometimes a Thr, and Asp/Glu in the heterotetramer anchor water-metal ion bridges. Absence of these residues leads to quinolone-resistance. Affinities of fluoroquinolones to topoisomerases are surprisingly selective although some sequence similarities between human- and bacterial type II topoisomerases exist. Selectivity is due to the facts that first the A and B subunits of human topoisomerases are fused, so that they function as homodimers. This structure is different from bacterial heterotetramers. Second, human type II topoisomerases lack the key residues anchoring the metal-ion bridges [328–332]. The highly selective mode of action of fluoroquinolones suggests that these agents may likely not affect mammals at clinically relevant concentrations, and if so, fluoroquinolones should interact with targets other than topoisomerases. However, high fluoroquinolone-concentrations ranging from 80 mg/L to 5.9 g/L inhibited eukaryotic type II topoisomerases, depleted mitochondrial DNA and inhibited mitochondrial functions [333–342].

DNA damage repair mechanisms are essential in pro- and eukaryotes in response to noxial events like UV-exposure. While bacteria repair DNA damages preferentially via the SOS response system [343, 344], the micro-RNA biogenesis system plays a role in DNA damage repair in eukaryotes apart from their essential impact on posttranscriptional regulation of gene expression modulating the RNA interference pathway [345–348]. The RNA interference pathway related endoribonucleases DICER and DROSHA promote DNA damage response activation by generating small non-coding RNAs with the sequence of the DNA flanking the double strand breaks. Micro-RNAs play also a role in various human diseases including carcinogenesis [348, 349]. From the quinolones tested ciprofloxacin, ofloxacin, norfloxacin, pipemidic acid, oxolininc acid, dilfloxacin, enoxacin [350], and also moxifloxacin [351] only norfloxacin [350], enoxacin [350, 352–355], pefloxacin [356] affected micro-RNA-, DICER- or DROSHA-activities thus exerting anti-neoplastic effects [357–360] (Table S1). Furthermore, downregulation of micro-RNA concentrations is associated with multiple forms of amyotrophic lateral sclerosis and also depression. The effect of enoxacin on neuromuscular function was examined in two mouse models in which micro-RNA biogenesis was enhanced and neuromuscular function was improved [361, 362]. Enoxacin reverted in vitro in human pluripotent stem cells the general micro-RNA downregulation related to ALS disease [363]. Enoxacin enhanced micro-RNA concentrations in vitro and in rat frontal cortex in animals with learned helpless behaviour as compared to animals with normal adaptive responses [364]. Thus, enoxacin and possibly other fluoroquinolones as well may ameliorate depressive behaviour.

Helicases share amino acid sequence- and enzyme activity homologies in bacteria, viruses, yeasts, and humans [365–367]. of the helicases contributing significantly to the genomic integrity in bacteria and humans is RecQ. Humans possess five RecQ helicases, i.e. RecQL1, − 4, − 5, WRN, and BLM. RecQL4 interacts with several replisome factors, amongst others with MCM2-7 [368]. Humans with RecQ mutations are likely to develop cancer and age prematurely [369]. Ciprofloxacin inhibited MCM2-7 [370]. RNA helicases have been implicated in proofreading processes. of these helicases, i.e. DHX9 has a key function in the regulation of biological processes, including tumorigenesis. Enoxacin inhibited DHX9 which is found in patients with lung- and prostate cancers [371, 372].

Ciprofloxacin, norfloxacin and enrofloxacin inhibited in HEK293 cells three α-ketoglutarate-dependent dioxygenases that require iron as a co-factor, which chelates with fluoroquinols [373]. Inhibition of α-ketoglutarate-dependent dioxygenases may on the hand explain fluoroquinol-induced nephrotoxicity and tendinopathy. On the other hand, fluoroquinol mediated dioxygenase inhibition lead unexpectedly to a reduction of hypoxia inducible transcription factor- (HIF)-1α and -2α concentrations due to inhibition of HIF mRNA transcription. Tumor hypoxia induces the up-regulation of genes associated with angiogenesis, glycolysis, adaptation to pH, and apoptosis via HIF-1α and HIF-2α, so that disruption of this pathway may be relevant in cancer therapy.

Various fluoroquinolones were found to exert anti-parasitic actions [10, 374–386] due to an interference with mitochondria derived organelles [269, 387–389]. Thus, patients with parasitic infections may benefit from fluoroquinolone treatment twofold: they are going to be treated not only of bacterial- but also of opportunistic parasitic infections, so that fluoroquinolone therapy should probably not be withdrawn upon elimination or exclusion of the bacterial pathogen.

## Conclusions and open questions

A synopsis of non-antimicrobial activities of antibiotics is provided in Table 2. In general, antibiotics interacted with multiple targets and exerted pleiotropic effects in eukaryotes. Many of these targets are multi-functional, so that one  specific effect cannot be attributed to one specific target and agent. In addition, different drug classes caused identical effects in eukaryotes. Thus, the anyhow complex modes of actions of antibiotics in eukaryotes may be even more multifaceted.

**Table 2:** Synopsis of non-antibacterial activities and modes of action of antibiotics in eukaryotes

Agent studied	Mode of action in eukaryotes	Effect recorded	Clinical efficacy*
Antibiotic class independent effects, inhibition of mitochondrial translation			
Aminoglycosides, Macrolides, Oxazolidinones, Chloramphenicol, Clindamycin	39S large mitosomal ribosome (63, 70–74, S65)	Mitochondrial dysfunction [63–68] Aminoglycosides [287] Oxazolidinones (68, 288, 289, S49) Tetracyclines (69, 287, S44 S66) Chloramphenicol (S42, S43)	Clinical trials have demonstrated a benefit from treatment of various cancers with aminoglycosides [223] and macrolides (76, S30-S35)
Tetracyclines, Tigecycline	28S small mitosomal ribosome [63, 295] 28S small mitosomal ribosome [78, 79]	Mitochondrial dysfunction (63–69, 287, S44, S66)	Clinical trials have demonstrated a benefit from treatment of various cancers with tetracyclines (S69–S71) The all-cause mortality was reduced in cancer patients prophylaxed or treated for infections with tigecycline versus patients receiving placebo or no antibacterial treatment [79, 405, 406]. Combination therapy with piperacillin/tazobactam increased the survival rate [407]
Rifampicin	Mitochondrial DNA-dependent RNA polymerase [72]	Reduced tumor progression due to suppression of delayed type hypersensitivity; proliferation of spleen lymphocytes (S2, S3)	n.st
Fluoroquinolones	Inhibition of mitochondrial topoisomerase 2α [333–342]	Mitochondrial dysfunction [333–342]	Clinical trials have demonstrated a benefit from treatment of various cancers with fluoroquinolones in treatment of various cancers [81, 82]
Bedaquillin	Mitochondrial ATP-synthesis (75, S109, S110)	Mitochondrial dysfunction (S109, S110)	n.st
ß-lactams	Inhibition of carnitine/acylcarnitine transporter, mitochondrial membrane (S6, S7)	Mitochondrial dysfunction (S6, S7)	n.st
Antibiotic class independent effects, inhibition of metallo-matrixproteinases			
Various	Formation of chelates or complexes with zinc and calcium Tetracyclines [87–89, 311–314] Macrolides [94–96] Vancomycin [91] ß-lactams [97–107] Bacitracin [92, 93] Isoniazid [89] Nitroxoline [108, 109]	Inhibition of MMP activity and/or reduction of gene expression in vitro Oxazolidinones (S128, S129) Tetracyclines (S111, S130–S187) Macrolides (S114–S127) Vancomycin (91, S198) ß-lactams (S111–S113) Bacitracin (S199) Isoniazid (S122, S195) Nitroxoline (S200, S201)	Doxycycline reduced MMP concentrations in patients with CF, COPD, and tuberculosis chronic wounds, abdominal aortic aneurysm, and ophthalmological diseases [116–134] Anti-neoplastic effects (S2, S3, S50-S56, S62) Commercially available for treatment of rosacea and periodontal diseases [136–142] Recovery of patients was associated with a decrease in MMP concentrations as a consequence of treatment of orthopaedic indications with ß-lactams [111, 112] and daptomycin [113], respiratory infections with macrolides [114, 115] Evidence confirming a clinical benefit resulting from MMP-inhibition in patients is lacking for the remaining agents
Fluoroquinolones Chloramphenicol	Formation of chelates or complexes with zinc and calcium [90, 110]	Increase of MMP concentrations Fluoroquinolones (S188-S193) Chloramphenicol (S44)	Adverse reactions
**S**ulfonamides			
2254 RP	Hyperplastic and trophic effects on the islets of Langerhans [6, 7, 21]	Hypoglycemia	Diabetes [6, 7, 21]
Sulfanilamide	Interaction of its thiol group with the Zn^2+^ ion in the homologous active centre of the carbonic anhydrase [153, 154]	pH regulation, HC0^−^_3_ reabsorption + secretion, production of aqueous humor + cerebrospinal fluid	Acid–base regulation, indirect functions in ureagenesis, gluconeogenesis, lipogenesis 153, 154)
ß-lactams			
Cefoperazone Ceftazidime	Inhibition of serine proteases [160, 161]	Inhibition of neutrophil elastase activity and prevention of α_1_-antitrypsin inactivation [160, 161]	n.st
7-aminocephalo-sporinic acid, Cephalosporin C, Cefotaxime, Ceftriaxone. Clavulanic acid, Sulbactam	Sequence homologies between PBPs and bacterial ß-lactamases and human metallo-ß-lactamases [166–168]	Inhibition of human metallo-ß-lactamases thus hypothetically interfering with inactivation of anti-neoplastic agents or tumorigenesis [178, 179]	n.st
Various ß-lactams	Sequence homologies between PBPs and DNA-polymerase-α [180–185]	Anti-neoplastic effects (S1-S10)	n.st
Cefonicid, Cefazolin, Cephalothin, Ampicillin, Piperacillin	Inhibition of mitochondrial carnitine/acylcarnitine transporter playing a pivotal role in tumors (S6, S7)	Inhibition of tumor cell growth in vitro (S6, S7)	n.st
Various ß-lactams, ceftriaxone in particular	Enhancement of gene expression uniquely of glutamate transporter GLT1 either through NF-κB mediated modulation of EAAT2 promotor activity, and/or due to chelation of copper [103, 106, 202–208]	Neuroprotectants in various models of neurological and neurodegenerative diseases, such as Alzheimer's disease, Parkinson, epilepsy, and strokes [103, 106, 186–208]	n.st
Various	covalent binding to cytokines or proteins [209–214]	Modulation of cytokine activity and regulation of gene expression in mammals [209–214]	n.st
Aminoglycosides			
Various	Interaction with 80S ribosomes and mitosomes [217–219] Induction of read-through of premature stop codons [220–222]	Induction of mutation suppression [220–226]	Clinical trials have demonstrated a benefit from treatment of patients with cystic fibrosis, Duchenne/Becker muscular dystrophy, ataxia telanlectasis, and patients with hemophilia A and –B [223–226]
Gentamycin Tobramycin	Inhibition of serine proteases [161]	Inhibition of neutrophil elastase activity and prevention of α_1_-antitrypsin inactivation [161]	n.st
Macrolides			
Various macrolides	Interaction with mitosomes Induction of read-through of premature stop codons [238, 242]	Induction of mutation suppression. Inhibition of proliferation in and promotion of apoptosis of human cancer cell lines	Reduction of tumor growth and lung metastasis Amelioration of the tumorigenic clinical symptoms caused by PTC mutations [248–253]
Clarithromycin	Not applicable. Clinical studies	Beneficial effects in patients with extranodal lymphoma of mucosa-associated lymphoid tissue, multiple myeloma, Waldenström’s macroglobulinemia, *H. pylori*-associated cancers, solid tumours like non-small- and small cell lung cancer, breast cancer (S30)	Clinical efficacy in patients with cancer and lymphoma, even as monotherapy (S30)
Erythromycin	The metabolite 8,9-anhydro-6,9-hemiketal serves as a motilin receptor agonist [254–259]	Prokinetic activity [254–259]	Increased gastrointestinal motility in patients with dysmotility [254–259]
Roxithromycin	The expression of gene *mocdc27* from the the fungus *Magnaporthe oryzae* is affected. This gene is highly homologous to *cdc27* playing a key role in colorectal cancer [260, 261]	In vitro inhibition of proliferation in and promotion of apoptosis of human cancer cell lines, thus confirming that CDC27 is targeted by roxithromycin [260, 261].	n.st
Phenicols			
Chloramphenicol	Interaction with mitosomes Induction of read-through of premature stop codons [242, 271–274]	In vitro inhibition of proliferation in and promotion of apoptosis of human cancer cell lines [242, 271–274]	n.st
Chloramphenicol	Inhibitio of appressorium formation in the fungus *Magnaporthe oryzae* because of an inhibition of MoDullard, a serine/threonine phosphatase [275]	MoDullard orthologs could be identified from the human genome, of which was inhibited by chloramphenicol [275]	n.st
Oxazolidinones			
Linezolid [286–289] Tedizolid [290–292]	Interaction with mitosomes Induction of read-through of premature stop codons Inhibition of phosphorylation of ERK1/2 which caused activation of a MAPK pathway member resulting in overexpression of mucin	Induction of mitochondrial dysfunction Supportive activity in treatment of respiratory diseases due to MMP-inhibition and reduced mucin production	n.st
Tetracyclines			
Various, doxycycline in particular	Interaction with 80S ribosomes and mitosomes [295, 296] Induction of read-through of premature stop codons [272, 273] Binding to double-stranded RNAs [20] Chelation with divalent cations [293, 294, 311–314]	Anti-proliferative effects on human cancer cell lines (S23, S44, S46, S57-S71) Antiparasitic effects 20, 307–312)	Tetracyclines were effective against various types of cancer even as monotherapy [11, 129–131] Favourable clinical outcomes have been observed in patients with orthopaedic diseases [297–304]
Fluoroquinolones			
Various	Inhibition of eukaryotic type II topoisomerases in cytoplasm and mitochondria 335–344) Interaction with DNA damage repair in eukaryotes via micro-RNA-, DICER- or DROSHA-activities [350–356] Inhibition of human RecQ helicases [370–372] Inhibition of hypoxia inducible transcription factor-1α [373] Chelation with divalent cations [90]	Antineoplastic effects [357–360] Amelioration of depressive behaviour [364] Antiparasitic effects [10, 374–389]	The all-cause mortality was reduced in cancer patients prophylaxed or treated for infections with fluoroquinolones versus patients receiving placebo or no treatment [80–82]

^*^ = It is important to note that evidence supporting clinical efficacies of the agents studied varies widely as most of the studies have been designed as retrospective observational studies rather than prospective randomized trials; furthermore, patient numbers were low in the majority of retrospective studies, thus affecting the quality of evidence. Numbers in parentheses indicate the references quoted; the prefix S refers to references quoted in the supplemental material; *n.st* not studied; *MMP* metallo-matrix-proteinases; *CF* cystic fibrosis; *COPD* chronic obstructive pulmonary disease

It is important to emphasize that the majority of studies reviewed above were planned as hypothesis generating preclinical experiments or retrospective observational clinical studies rather than prospective, randomized clinical trials, so that evidence supporting these potential novel indications varies widely. But still these data demonstrate that antibiotics interact with eukaryotic targets, so that therapeutic options of an antibiotic treatment may likely be more diverse than expected. Although none of the antibiotics should be used at the time being for monotherapy of non-infectious diseases, they could be used as adjunctive therapeutics in cancer patients or patients with parasitic diseases. Macrolides, tetracyclines, aminoglycosides, and fluoroquinolones have been shown to be clinically effective as anti-neoplastic agents, even in monotherapy, and were found to synergise with cancer drugs. Therefore, antibiotics could possibly be used not only for prophylaxis- or treatment of bacterial infections, but also for adjuvant therapy in cancer patients. Treatment modalities could hypothetically be individualized by selecting agents for prophylaxis or treatment of bacterial infections which are active against the specific cancer type and which synergize with the specific cancer drug administered to the patient. Likewise, tetracyclines and fluoroquinolones exerting anti-parasitic activities should probably not be withdrawn upon elimination or exclusion of the bacterial pathogen but should be continued in parallel to administration of the specific anti-parasitic agent. Their activities against RNA-viruses [10–12] could complement the spectrum of activities. Several additional targets for antibiotics in human cells have been identified (Table 2) apart from mitochondrial functions and MMPs. This may open up new opportunities for the focused evaluation of non-antimicrobial activities of antibiotics.

However, following effect dichotomies have to be considered: antibiotics could be used as adjunctive therapeutics but long-term use may possibly promote resistance development, tumorigenesis, and obesity. Antibiotics were used in some of the indications for several weeks and even months and treatment of genetic disorders may last for life. Toxicological implications of long term antibacterial treatment have not yet been assessed with the majority of antibiotics although it is documented that prolonged use of linezolid and chloramphenicol was associated with adverse events. The impact on the microbiome and resistance development has also not been analysed in these studies. Systematic reviews have revealed that increased consumption of antibiotics was associated with resistance development at the individual patient level and also in the community. Moreover, antibiotic-resistance was not always, but usually, associated with a significant economic burden resulting from (re-)admission to hospital, need for i.v.-administration, or even use of a less well tolerated antibiotic [390–396]. Antibiotics causing mitochondrial dysfunction may promote tumorigenesis [397–400], obesity [401], and psychiatric disorders [402, 403]. Furthermore, this review has demonstrated that antibiotics may have an unpredictable impact on cell culture metabolism, gene expression and signalling cascades thus supporting a previous study entitled “are cell culture data skewed?” [404]. These open questions should be carefully considered at the present time and be addressed henceforth.

The effects of antibiotics on eukaryotes are due to identical mechanisms as their antibacterial activities because of structural and functional homologies of pro- and eukaryotic targets, so that the effects of antibiotics on mammals are integral parts of their overall mechanisms of action. A purposeful use of antibiotics not only as antibacterial agents but also as agents targeting the human body and his functions should be considered.

## Addendum search strategy

Publications addressing four topics were screened: first, impact of antibiotics on growth of eukaryotic/mammalian cells/cell cultures. Second, impact of antibiotics on mitochondrial functions, eukaryotic translation/transcription, and/or enzyme activities like metallo-matrix-proteinases, serine proteases, etc., read-through, premature stop codons. Third, interaction with mitosomes, 80S ribosome, RNA, DNA. Fourth, interaction of antibiotics with eukaryotic targets. Search strategy and selection criteria were based on the combination of key words sulphonamides, ß-lactams, aminoglycosides, macrolides, chloramphenicol, oxazolidinones, tetracyclines, fluoroquinolones, the corresponding single agents of these drug-classes, and antibiotics in general. Growth inhibition, tumor growth, inhibitory concentration IC_50_, homologous, orthologs, paralogs, chelation, complexation. Articles summarized in recent reviews were excluded from this synopsis and the reviews are quoted instead.

## Electronic supplementary material

Below is the link to the electronic supplementary material.

Supplementary file1 (DOCX 293 kb)
