# Supplementary material for: Selective toxicity of antibacterial agents—still a valid concept or do we miss chances and ignore risks?
Source: Infection. 2020 Dec 23;49(1):29–56. doi: 10.1007/s15010-020-01536-y (PMC7851017; doi:10.1007/s15010-020-01536-y)
Supplement: Supplementary file 1 — Supplementary file1 (DOCX 293 kb) [file 15010_2020_1536_MOESM1_ESM.docx]

Table S1

Anti-neoplastic effects of and inhibition of mitochondrial functions by antibiotics (CPX=ciprofloxacin; GEN=gentamicin; TOB=tobramycin; AMK=amikacin; NEO=neomycin; KAN=kanamycin; SIS=sisomycin; LIV=lividomycin, PAR=paromomycin, HYGB=hygromycinB; ERY=erythromycin; RXM=roxithromycin; AZM= azithromycin; CLR= clarithromycin; DTM=dirithromycin; -JSM=josamycin; DOX = doxycycline; MIN = minocycline; CHL=chloramphenicol; AMP=ampicillin, TET=tetracyclin; TEL=telithromycin; CIP=ciprofloxacin; LVX=levofloxacin; ENX=enoxacin; FLE=fleroxacin; GAT=gatifloxacin; GRX=grepafloxacin; LOM=lomefloxacin; MXF=moxifloxacin; NAL=nalidixin; NOR=norfloxacin; OFX=ofloxacin; SPX=sparfloxacin; TVA=trovafloxacin; PFX=pefloxacin; PTC=premature termination codon; CSC=cancer stem cell; MMP= metallo-matrix-proteinase)

| Agent | Model | Effective conc. | Target, aim of the study | Effect, comment | ref |
| --- | --- | --- | --- | --- | --- |
| **sulphonamides** | | | | | |
| Sulfamethoxazol in combination with trimethoprim | 3 human bladder cancer cell lines exposed to up to 1,000mg/L for 96h | Range of significant cytotoxicity 31.2-62.5mg/L | Evaluation of the cytotoxicity of commonly used urinary antibiotics at clinically relevant concentrations | Administration of antibiotics after resection of bladder tumors might prevent seeding of cancer cells thus decreasing the recurrence rate | 1 |
| **ß-lactams** | | | | | |
| Penicillin G | HeLa, K562 leukemic cell line; exposure for 6h + 12h | 7.5 + 10% statistically significant inhibition of cell growth | Non antibacterial ß-lactam derivatives induced apoptosis in cancer cells. Doesn’t penicillin G really not do that? | Growth of cancer- but not of normal cells were inhibited by relatively high penicillin G concentrations. | 2 |
| PenicillinG, Piperacillin, Mezlocillin,  Cefalothin, Cefamandole, Cefotaxime, | BALB/c-mouse-sarcoma L-1;  In vitro cell lines: human lung cancer E-14, human malignant melanoma MEW | 7- to 10 days treatment,  human doses on a mg/kg/day basis | Interest in „immunomodulatory“ functions of antibiotics in general, so that  immune functions,  tumor growth,  intestinal flora,  were analyzed | Tumor suppression only by mezlocillin and piperacillin. Complete eradication of aerobic and anaerobic flora – associated with inhibition of local tumor growth. Suppression of delayed type hypersensitivity + of spleen lymphocyte proliferation. No in vitro activity | 3-5 |
| Cefazolin | 3 human bladder cancer cell lines exposed to up to 5,000mg/L for 96h | Range of significant cytotoxicity 19.5-156.3mg/L | Evaluation of the cytotoxicity of commonly used urinary antibiotics at clinically relevant concentrations | Administration of antibiotics after resection of bladder tumors might prevent seeding of cancer cells thus decreasing the recurrence rate | 1 |
| Cefonicid,  Cefazolin, Cephalothin, Ampicillin, Piperacillin | Mitochondrial carnitine/acylcarnitine transporter | IC_50_ in g/L  Cefonicid = 3.72  Ampicillin = 2.65  All others >4.00 | carnitine/acylcarnitine transporter playing a pivotal role in tumors | The reversible IC_50_ are higher than relevant plasma concentrations | 6, 7 |
| Cephalexin | *Patients*, bladder cancer vs. CPX | 3 days treatment | Prevention of recurrence of cancer | Study completed 2007; no results published | 8 |
| Ceftriaxone | A549, H520 and H1650 lung cancer cells.  Tumor cells implanted into the flank of nude mice. | Ceftriaxone (500 μM) inhibited anchorage-independent in vitro- and in vivo growth of A549, H520 and H1650 lung cancer cells.  Ceftriaxone directly bound with Aurora B and suppressed its activity in vitro and in vivo. | Other cephalosporins inhibited tumor growth but ceftriaxone had not yet been studied. | Ceftriaxone directly bound to Aurora B - a chromosomal passenger protein critical for accurate chromosomal segregation, cytokinesis and regulation of the mitotic checkpoint | 9 |
| Ceftiofur | Pre-treatment of RAW 264.7 mouse macrophage cells  with 1, 5, 10mg/L 1h prior to LPS stimulus | Significantly inhibition of phosphorylation of extracellular signal-regulated kinases, p38 and c-jun NH2-terminal kinases (JNKs) in RAW264.7 . Downregulation of cytokine production. | Analysis of the immunomodulatory potential of ceftiofur | Ceftiofur exerted immunomodulatory activities and interfered with signal transduction. Tumor necrosis factor α, intereukins 1ß and 6, but not Il-10 and extracellular signal-regulated kinases p38, c-jun NH2-terminal kinase phosphorylation, and p65-NF-κB translocation were downregulated | 10 |
| **Aminoglycosides** | | | | | |
| Gentamicin | 5 lymphoma cells | 90mg/L | Examination if eukaryotic translation may be affected or not. | Inhibition of cell growth, arrest of the cells in the G1 phase of the cell cycle, | 11 |
| Gentamicin | HeLa cells, mitochondrial delivery | at an extracellular conc. of 4.77g/L the mitochondrial occupation rate was about 2.5% | Use of a liposomal nanocarrier for mitochondrial delivery resulting in high intracellular concentrations. | Mitochondrial disorder, strong cytotoxic effect | 12 |
| Gentamicin | Skin fibroblasts | 200-500mg/L | Read-through of PTC; Stüve-Wiedemann Syndrome | Partial restauration of protein synthesis | 13 |
| Gentamicin | 12 different human cancer cell lines.  Quantification of 50% growth inhibition (GI_50_) or 50% lethal concentration (LC_50_) | GI_50_ = 2.7mg/L against pancreatic cell line and  GI_50_=<1mg/L against human bladder carcinoma cell line.  The remaining cell lines were inhibited by concentrations >20mg/L | Repositioning of old drugs |  | 14 |
| Gentamicin, Paromomycin, Geneticin | xenograft experiments, 1 human embryonic kidney- and 3 human colon carcinoma cell lines, and the Apc ^Min/+^ mouse model | Statistically significant induction of read-through (in mg/mL)  GEN 0.75 + 1.0  PAR 0.5, 0.75, 1.0  geneticin 0.5, 0.75, 1.0 | 30% of patients with sporadic and hereditary colorectal cancer carry PTC mutations in the gene encoding the multifunctional tumour suppressor protein adenomatous polyposis coli | These agents induced read-through of PTCs at low concentrations and ameliorated the tumorigenic clinical symptoms caused by PTC mutations in the APC gene | 15 |
| Gentamicin ,  Amikacin,  Paromomycin | Increased read-through of the MoMLVgag–pol junction beyond the normal levels of recoding induced by the pseudoknot, and virus production | Statistically significant induction of read-through (in mg/mL)  GEN 25, 50  AMK 1.5, 3.0  PAR 0.5, 1.0 | Effects of read-through on eukaryotic recoding in the context of retroviral read-through sequences in a murine leukemia virus model | Drug-induced increases in read-through resulted in a reduced production of infectious leukemia virus particles. | 16 |
| Gentamicin  Geneticin  amikacin | Mutationally inactivated tumor suppressor gene *TP53* in a human cancer cell line | Gentamicin 800mg/mL  Geneticin 50, 100, 200 mg/mL  Amikacin 2 mg/mL | Read-through of the p53 tumor suppressor gene which is mutated in >50% of all humans for which 8% of the mutations are non-sense mutations | Induction of read-through and expression of full length p53.  Decreased viability of cancer cells even in the absence, but in particular the presnece of nonsense-mutated p53 gene | 17, 18 |
| Gentamicin | Read-through for 66 sequences containing a stop codon including APC- and p53 genes in cultured mammalian cells. | 800mg/L | The readthrough level depends on the nature of the stop codon and the surrounding nucleotide context, but little was known about the mechanisms of readthrough and responses to aminoglycosides. In this study, a large set of nonsense mutations for an in-depth statistical analysis designed to decipher the element of the nucleotide context responsible for modulating readthrough levels was used. | Efficiency of read-through after treatment is determined by the complex interplay between the stop codon and a larger sequence context. The nucleotide immediately upstream from the stop codon was a major determinant of gentamicin response | 19 |
| GEN, TOB, AMK, NEO, KAN, SIS, LIV, PAR,  HYGB, geneticin | Reviews.  Various in vivo models, and in particular  *patients* | Patients with cystic fibrosis, Duchenne/Becker muscular dystrophy, ataxia telanlectasis, and patients with hemophilia A and –B benefitted from treatment with standard doses. | reviews | Demonstration of in vivo and in particular clinical relevance of aminoglycoside-induced read-through of PTCs | 20-22 |
| **macrolides** | | | | | |
| Josamycin, Spiromycin | xenograft experiments, 1 human embryonic kidney- and 3 human colon carcinoma cell lines, and the Apc ^Min/+^ mouse model | Statistically significant induction of read-through (in mg/mL)  Josamycin 0.1, 1.0, 10  Spiromycin 0.3, 3.0 | 30% of patients with sporadic and hereditary colorectal cancer carry PTC mutations in the gene encoding the multifunctional tumour suppressor protein adenomatous polyposis coli | These compounds ameliorated the tumorigenic clinical symptoms caused by PTC mutations in the APC gene | 15 |
| Erythromycin  Azithromycin | Incubation of MCF-7 breast cancer stemm cells (CSC) in normoxic (20% O_2_) and hypoxic (4% O_2_) conditions for 14 days | Erythromycin inhibited CSC 62.5 % in normoxia vs. 58.9% in hypoxia;  azithromycin was not inhibitory | Impact of tumor microenvironment, i.e. hypoxia, playing a significant role in maintaining cancer stem cell population on drug efficacy | The anti-tumor effect of erythromycin is not significantly affected by microenvironemental factors. Only 14-membered macrolides may likely exhibit anti-neoplastic acivities. | 23 |
| Erythromycin  Roxithromycin | mouse B16F10 melanoma cell line inoculated s.c. into the flank of mice | Significant growth inhibition at ERY doses of 10 mg/kg or 50 mg/kg and RXM doses of  10 mg/kg but not 50 mg/kg | Confirmatory study monitoring cancer growth. | Approximately 50% tumor growth inhibition by ERY and RXM. Others quoted by the authors confirmed these data and showed that RXM reduced lung metastasis. ERY and RXM synergize with anti-cancer agents | 24 |
| Erythromycin | Tumor bearing mice in both allogenic and syngenic mice dosed with 1-10 mg/kg/day | Maximum effect achieved at 5mg/kd/day | Confirmatory study monitoring tumor growth and cellular immunity | Erythromycin exhibited an indirect antineoplastic activity by enhancing the production of IL-4 which augmented the tumoricidal activity of macrophages | 25 |
| Erythromycin  Clarithromycin | acute leukemia cells, both myeloid and lymphoid. | Erythromycin LD_50_=57.25mg/L,  clarithromycin LD_50_=41.89mg/L | Inhibition of hERG1 potassium channels has antileukemic effects. | Antileukemic activity depended on a modulation of both autophagy and intracellular signaling pathways; these effects were mediated by hERG1 channels. | 26 |
| Josamycin  Spiromycin | Xenograft experiments, 1 human embryonic kidney- and 3 human colon carcinoma cell lines, and the Apc ^Min/+^ mouse model | Statistically significant induction of read-through (in mg/mL)  Josamycin 0.1, 1.0, 10.0  Spiromycin 0.3, 3.0 | 30% of patients with sporadic and hereditary colorectal cancer carry PTC mutations in the gene encoding the multifunctional tumour suppressor protein adenomatous polyposis coli | The two agents induced read-through of PTCs at low concentrations and ameliorated the tumorigenic clinical symptoms caused by PTC mutations in the APC gene | 15 |
| Roxithromycin | Review | Review | Before the background that in the fungus *Magnaporthe oryzae* RXM interacts with the CDC27 homolog MoCDC27 five studies confirming this hypothesis are quoted | Roxithromycin inhibited proliferation in and promoted apatosis of human lymphocytes and exhibited an anti-tumor effect ( review of five studies) | 27 |
| Roxithromycin, Erythromycin, Flurithromycin, Dirithromycin,  Clarithromycin,  Azithromycin | Human aldosterone-producing adrenocortical cancer cell lines | Macrolides selectively inhibited KCNJ5^MUT^ but not KCNJ5^WT^; IC_50_ (mg/L):  RXM=0.184  ERY=7.728  FLM=no fit  DTM=13.194  CLR=0.531  AZM=4.262 | Mutations in the potassium channel KCNJ5 ( KCNJ5^MUT^) account for 50% of aldosterone producing adenomas. Inhibition of KCNJ5^MUT^ induced induction of aldosterone synthesis | Macrolides had the potential for treatment of aldosterone-producing adenomas harbouring KCNJ5^MUT^.  IC_50_ values are higher than the mean maximal plasma concentrations following p.o. dosing apart from RXM and ERY. | 28 |
| Roxithromycin  Clarithromycin,  Josamycin, Azithromycin | Mouse (C57BL mice) dorsal air sac model of angiogenesis.  Implantation of chambers containing B16BL6 melanoma cells | Intraperitoneal injection of 50mg/kg/day reduced tumors following RXM treatment by 41%, and CLM by 56%;  azithromycin and josamycin had no effect | Reduction of tumor size and suppression of pulmonary metastasis may be dependent from the 14-, 15, and 16-membered macrolide structure. | Only the 14-membered macrolides RXM + CLR may have an antiangiogenic and antitumor effect; 15- and 16-membered AZM and JSM, respectively, were ineffective.  See above, reference 23. | 29 |
| Clarithromycin | Review of preclinical and in particular clinical data | review | Clinical efficacy in patients with multiple myeloma, Waldenström’s macroglobulinemia, some *H. pylori*-associated cancers, solid tumours like non-small- and small cell lung cancer, breast cancer | Clarithromycin was clinically effective in the treatment of cancer and lymphoma, even as a single treatment.  Clarithromycin was effective in combination with other treatments in early stage multiple myeloma, and Waldenström macroglobulinemia. | 30 |
| Clarithromycin | *Patients* with extranodal lymphoma of mucosa-associated lymphoid tissue | 500mg b.i.d. for 6 months;  3 courses of 500mg b.i.d. , days 1-21 days, every 35 days;  4 courses of 1,000mg b.i.d., days 1-14, every 21 days | Clinical study on the dose-effect relationship of clarithromycin monotherapy in cancer patients. | Overall response rate = 52%;  Two year pregression free interval = 56% | 31-33 |
| Clarithromycin | *Patients* with advanced multiple myeloma (n=56) | 500mg b.i.d. for 5 months single treatment | Clinical study on long-term treatment with clarithromycin as monotherapy. | Three patients (5.36%) had an objective or minor response. | 34, 35 |
| Clarithromycin  Azithromycin | Comparison of the effect on autophagy inhibition.  Enhancement of gefitinib-induced cytotoxic effect on pancreatic cancer cell lines BxPC-3 and PANC-1 | 5-100μM clarithromycin or azithromycin +/- 25μM gefitinib | Use of macrolides as ‘chemosensitizers’ for epidermal growth factor receptor tyrosine kinase inhibitor therapy in pancreatic cancer patients to enhance non-apoptotic tumor cell death induction. | Enhanced gefitinib-induced cytotoxicity in pancreatic cancer cell lines. The efficiency of macrolides in blocking autophagy flux correlated well with their enhancement of gefitinib-induced cytotoxicity.  Azithromycin was more active than clarithromycin | 36 |
| Clarithromycin  Azithromycin | Multiple myeloma cell lines IM-9, U266 and RPMI8226 | IC_50_ of bortezumib alone ranged from 7.2 nM to 12.2 nM. IC_50_ decreased >4-fold in combination with 25 or 50 mg/L of either macrolide | Possibility of using a macrolide in combination with a proteasome inhibitor for therapy of multiple myeloma. | Azithromycin, clarithromycin and erythromycin blocked autophagy flux. Combined treatment of bortezumib and azithromycin or clarithromycin enhanced cytotoxicity in multiple myeloma cell lines, although exposure to either agent alone exhibited almost no cytotoxicity | 37 |
| Clarithromycin  Azithromycin | Squamous cell carcinoma cellline CAL27,  human pharyngeal carcinoma cell line Detroit 562 | 50μM of each macrolide | Clarithromycin and azithromycin may be used as inhibitors of autophagy flux. However, mechanisms of macrolide-induced autophagy inhibition are unknown | Clarithromycin and azithromycin exerted cytotoxic effects on head and neck squamous cell carcinoma cell lines only under aminoacid-depleted culture conditions.  Macrolides blocked autophagy flux and induced cell death via induction of transcription factor CHOP only in amino acid depleted media, so that macrolides may be useful for “tumor- starvation” . | 38 |
| Azithromycin | Colon cancer cell lines HCT‑116, SW480, SW620 and DiFi | 25-225μM azithromycin,  15.625, 100, 50 nmol/L Tumor necrosis factor‑related apoptosis‑inducing ligand | Synergism of azithromycin with tumor necrosis factor‑related apoptosis‑inducing ligand, which selectively targets tumor cells without damaging healthy cells. | Azithromycin increased the expression of death receptors and blocked autophagy flux in colon cancer cells. Azithromycin treatment enhanced tumor necrosis factor‑related apoptosis‑inducing ligand-induced apoptosis via the up-regulation of DR4 and DR5 in colon cancer cells. | 39 |
| Azithromycin | HeLa, SGC-7901 and BHK-21 cells | IC_50_ of azithromycin alone were15.66, 26.05 and 91.00mg/L at 72h post incubation. In combination with vincristine the IC_50_ of azithromycin were reduced to 9.47μg/mL, 8.43 μg/mLand 40.15μg/mL | Anti-proliferative and anticancer activity of azithromycin +/- vincristine | Azithromycin inhibited proliferation and induced apoptosis in cervical- and gastric cancer cells. Azithromycin in combination with vincristine exhibited a selective synergistic effect on cancer cell apoptosis, partly through caspase activation, i.e. intrinsic mitochondrial apoptotic pathway. | 40 |
| Azithromycin | 6 lung tumor associated endothelial cells (HTL-EC) | 1, 5, 10 μM azithromycin +/- endothelial growth factor receptor 2 | Vascular endothelial growth factor receptor 2 mediated focal adhesion and phosphoinositide-3-kinase/protein kinase B signaling pathways in human lung tumor associated-endothelial cells | Azithromycin inhibited HLT‑EC adhesion and vascular endothelial growth factor‑induced proliferation of HLT‑ECs in a dose‑dependent manner. In addition, azithromycin induced apoptosis of HLT‑ECs even in the presence of vascular endothelial growth factor.  Azithromycin inhibited proliferation and induced apoptosis in multiple lung cancer cell lines. | 41 |
| Azithromycin | 12 different human cancer cell lines.  Quantification of 50% growth inhibition (GI_50_) or 50% lethal concentration (LC_50_) | GI_50_=2.9mg/L against bladder cancer.  The remaining 11 cell lines were inhibited by concentrations >4mg/L | Repositioning of old drugs | Azithromycin was an effective growth inhibitor, but was not lethal. These studies (14, 23, 28, 36-41) with AZM in various different models demonstrated in contrast to data generated in ref’s 23 + 29, that AZM affected cancer cell growth. | 14 |
| **Phenicols** | | | | | |
| Chloramphenicol | Incubation of MCF-7 breast cancer stemm cells in normoxic (20% O_2_) and hypoxic (4% O_2_) conditions for 14 days |  | Impact of tumor microenvironment, i.e. hypoxia, playing a significant role in maintaining cancer stem cell population on drug efficacy | Chloramphenicol inhibited cancer stemm cells to 71.5% under normoxic- vs. 73.7% under hypoxic conditions | 23 |
| Chloramphenicol | RPMI8266 and U266 cells, as well as unstimulated freshly isolated peripheral blood mononuclear cells | IC_50_ = 142.45 and 315.22mg/L for RPMI8266 and U266, respectively. Reduction of ATP levels at ≥ 25mg/L, inhibition of cell growth at ≥ 25-50mg/L, induction of myeloma cell apoptosis at ≥ 50mg/L. | Verification/falsification of the theory that suppression of mitochondrial ATP synthesis may reduce tumor growth | Chloramphenicol increased levels of cytochrome c, cleaved caspase-9 and cleaved caspase-3, so that myeloma cell apoptosis occurs via the mitochondria-mediated apoptosis pathway. However, IC_50_ values exceed achievable serum concentrations amounting to 5-9mg/L 1-2 h following i.v. injection of 500mg and to 10-30mg/L following p.o. administration of 1g | 42 |
| Chloramphenicol | K562 erythroleukemia cells.  Exposition to 10mg/L CHL for 4 days | Significant decreases in  -cell surface transferrin receptor expression  -de novo ferritin synthesis  -cytochrome *c* -oxidase activity,  -ATP levels,  -respiratory activity, -cell growth | Effect of chloramphenicol on  1. mitochondrial function.  2. two key proteins in iron metabolism: the transferrin receptor, which mediates iron uptake, and ferritin, the iron storage protein.  Thus, these studies may contribute to explain the mechanisms by which CHL impairs erythropoiesis and heme synthesis and affects mitochondrial functions. | Chloramphenicol interfered with mitochondrial functions resulting in significantly reduced growth of the erythroleukemia cell line and decreased expression of iron transport proteins. Iron is an essential fro the activity of ribonucleotide reductase and thus DNA synthesis. Iron depletion affects the electrontransport chain resulting in reduced ATP synthesis. | 43 |
| Chloramphenicol | H1299cells + 10-100mg/L chloramphenicol in conditioned medium | CHL activated phosphatidylinositol 3-kinase /Akt/c-Jun N-terminal kinases signaling and increased nuclear c-Jun activity, which contribute to MMP-13 expression and H1299 cell invasion.  Cellular levels of thep21waf1/cip1protein and p21waf1/cip1mRNA were in-creased through a p53-independent pathway, possiblybecause of the stabilization of p21waf1/cip1mRNA inchloramphenicol-treated cells. | Verification/falsification of the hypothesis that CHL induces senescence-like responses, especially the mitochondria-to-nucleus stress signals and MMP-expression in CHL-exposed H1299 cells.  Other mitochondrial translation inhibitors (tetracyclines, clindamycin) served as comparators. | Chloramphenicol might trigger a novel mitochondrial stress signal that results in an increase of cancer cell invasion and an acceleration of cancer progression.  Tetracyclines and clindamycin caused analogues effects.  Chloramphenicol rendered the cells resistant to mitomycin-induced apoptosis. | 44, 45 |
| Chloramphenicol | 3 human Burkitt’s lymphoma cell lines, 1 ovarian serous adenocarcinoma, 1embryonic kidney cell line | Cytotoxicity in all 5 cell lines EC_50_ 0.6 to 1.4 mM | Inhibition of mitochondrial functions by protein synthesis inhibiting antibiotics may in theory augment the activities of recombinant immunotoxins developed for cancer treatment. | EC_50_ values were well above achievable plasma concentrations of approximately 15-46μM . But nevertheless, chloramphenicol showed synergism with recombinant immunotoxins | 46 |
| **Oxazolidinones** | | | | | |
| Linezolid | 3 human Burkitt’s lymphoma cell lines, 1 ovarian serous adenocarcinoma, 1embryonic kidney cell line | Cytotoxicity in embryonic kidney cell line only, EC_50_ 0.5mM | Inhibition of mitochondrial functions by protein synthesis inhibiting antibiotics may in theory augment the activities of recombinant immunotoxins developed for cancer treatment. | EC50 values were well above achievable plasma concentrations of approximately 1-12μM . No synergism with recombinant immunotoxins could be demonstated. | 46 |
| Linezolid | Cancer stem cells,  chemoresistant cells derived from triple negative breast cancer cells.  Xenograft model in NMRI mice inoculated with three tumor cell lines. One concentration of 100mg/L was used in vitro, anumals were treated with 100mg/kg/day | Linezolid decreased mitochondrial functions,  suppressed cancer cell growth, increased apoptosis. | Verification/falsification of the theory that antibiotics inhibiting translation of mitoribosomes and inducing mitochondrial dysfunction exert anti-cancer activities. | Linezolid (and hygromycin) induced mitochondrial dysfunction.  Linezolid in combination with an autophagy blocker augmented tumor cell death. | 47, 48 |
| Tedizolid | In vitro myeloid cell line and mouse model of leukemia | Tedizolid overcame venetoclax resistance (an inhibitor of B cell leukemia 2). Combination of both drugs augmented integrated stress response, which, in turn, suppressed glycolytic capacity, resulting in adenosine triphosphate depletion and subsequent cell death. | Ribosome-targeting antibiotics can help overcome venetoclax resistance by suppressing mitochondrial respiration and activating the cellular stress response. | Inhibition of mitochondrial translation is an effective approach to overcoming venetoclax resistance. Combination treatment with tedizolid and venetoclax was superior to either agent alone in reducing leukemic burden. | 49 |
| **tetracyclines** | | | | | |
| Tetracyclines | reviews | Reviews | Therapeutic potential of mitochondrial translation inhibition for treatment of various cancers | Tetracycline monotherapy is active against various types of cancer | 50-56 |
| Doxycycline | Breast- and prostate cancer cell lines | Inhibition of cell proliferation in the nM to μM range | Mitochondrial targeting is associated with an anti-cancer effect | Doxycycline exerted effects in cancer cells only .  Doxycycline synergized with doxorubicin | 57-61 |
| Doxycycline | Breast cancer *patients* | 200mg/day for 14 days before surgery | Assessment of the clinical relevance of suppression  of key mitochondria-related proteins and inhibition of mitochondrial functions | Post treatment samples demonstrated a statistically significant decrease in in the stemness marker CD44 as compared to pre-doxycyline samples. | 62 |
| Tetracycline | 3 human Burkitt’s lymphoma cell lines, 1 ovarian serous adenocarcinoma, 1embryonic kidney cell line | Cytotoxicity in ovarian serous adenocarcinoma and embryonic kidney cell line only, EC_50_ 0.6->1.0mM | Inhibition of mitochondrial functions by protein synthesis inhibiting antibiotics may in theory augment the activities of recombinant immunotoxins developed for cancer treatment. | EC_50_ values were well above achievable plasma concentrations of approximately 2-23μM . No synergism with recombinant immunotoxins could be demonstrated. | 46 |
| Tetracycline, Doxycycline | Incubation of MCF-7 breast cancer stemm cells in normoxic (20% O_2_) and hypoxic (4% O_2_) conditions for 14 days |  | Impact of tumor microenvironment, i.e. hypoxia, in maintaining cancer stem cell population, and on drug efficacy. | Tetracycline inhibited CSC 56.0% in normoxia vs. 65.0% in hypoxia.  Doxycycline inhibited CSC 75.2% in normoxia vs. 68.5% in hypoxia.  Doxycycline overcomes hypoxia induced resistance to anti-angiogenic agents. | 23, 62, 63 |
| Doxycycline | BALB/c-mouse-sarcoma L-1; | 7- to 10 days treatment,  human doses on a mg/kg/day basis | Role of immune functions in cancer therapy. | Tumor suppression. Suppression of delayed type hypersensitivity + of spleen lymphocyte proliferation. | 2, 3 |
| Doxycycline,  Minocycline | H1299cells + 10mg/L DOX- or MIN in conditioned medium | Doxycycline and minocycline inhibited mitochondrial translation, reduced ATP concentrations by >50%, induced MMP-13 expression 1.5- to 2-fold | Verification/falsification of the hypothesis that tetracyclines induce senescence-like responses, especially the mitochondria-to-nucleus stress signals and MMP-expression in chloramphenicol-treated H1299 cells.  Other mitochondrial translation inhibitors served as comparators. | Tetracyclines might trigger a novel mitochondrial stress signal that results in an increase of cancer cell invasion and an acceleration of cancer progression.  Chloramphenicol and clindamycin caused analogous effects. | 44 |
| Doxycycline,  Minocycline | Human acute myeloid leukemia HL-60 cell line.  0.5 to 100mg/L doxy- or minocycline | Loss of viability, IC_50_  of doxycycline = 9.2mg/L  of minocycline = 9.9mg/L  Induction DNA double strand breaks.  Loss of mitochondrial membrane potential. Caspase activation. | Verification/falsification of the anti-neoplastic activities of teracyclines | Doycycline and minocycline exerted anti-proliferative and pro-apoptotic effects at clinically achievable concentrations. | 64 |
| Doxycycline | Breast cancer stem cells. Analysis of DNA-PK, an enzyme involved in DNA repair and also in radio-resistance | Doxycycline reduced DNA-PK expression significantly and radiosensitized cancer stem cells | Verification/falsification if DNA damage response may represent another target for doxycycline in cancer stem cells | Doxycycline is a potent DNA-PK inhibitor as well as radiosensitizer | 65, 66 |
| Doxycycline | HeLa- and small cell lung cancer cells | 0.5mg/L doxycycline exhibited anti-apoptotic effects by inhibiting caspase-3 and -8 activation in HeLa cells, but 5.0, 10, 20 mg/L doxycycline induced apoptosis and expression of caspases in lung cancer cells | Carcinogenesis is associated with the deregulated activity of cysteine-aspartic proteases known as caspases, indicating the involvement of an intrinsic mitochondrial apoptotic pathway. Doxycycline may interfere with caspase activity. | Proliferation of both cell lines was inhibited by doxycycline, however the effects on caspases were contradictory | 67, 68 |
| Doxycycline | 12 different human cancer cell lines.  Quantification of 50% growth inhibition (GI_50_) or 50% lethal concentration (LC_50_) | GI_50_=6.2 to 9.0mg/L against ovarian cancer  GI_50_=2.4mg/L against bladder cancer  GI_50_=7.2 mg/L against cervical cancer,  GI_50_=3.4mg/L against pancreatic cancer  GI_50_=5.2mg/L against breast cancer  GI_50_=4.6mg/L against lung cancer | Repositioning of old drugs |  | 14 |
| Tigecycline | Reviews  Various cell lines:  acute myeloid leukemic-, non-small cell lung cancer-, gastric cancer- hepatocellular carcinoma-, breast cancer-, melanoma-, cervical squamous cell carcinoma-, and glioblastoma cell lines  Completed clinical studies in “cancer”, haematological disorders, acute lymphoblastic leukemia. | Various.  Response rates in treatment of „cancer“=48-64%,  hematological malignancies=43%,  acute lymphoblastic leukemia=not available.  Marked anti-proliferative effects in vitro. | Anti-tumor activity of tigecycline was observed in the course of clinical studies on the antibacterial activies in e.g. skin- and soft tissue infections. Thus, the question has been addressed if tigecycline may target multiple cancer types. | Tigecycline exerted its therapeutic effects via inhibition of mitochondrial functionality, interference with various signal transduction pathways, and synergized with anti-neoplastic agents.  However, myelotoxicity was recorded in bone marrow transplant patients (2 out of 2) and others terminated tigecycline administration prematurely because of intolerable side effects.  The FDA issued a warning concerning an increase in all cause mortality in tigecycline treated patients versus comparator in randomized controlled trials. | 69-71 |
| **Fluoroquinolones** | | | | | |
| CIP, LVX, ENX, FLE, GAT, GRX, LOM, MXF, NAL, NOR, OFX, SPX, TVA, PFX | Reviews; various cancer cell lines | Apoptosis; S/G_2_ phase arrest; inhibition of proliferation; synergism with anti-neoplastic agents at clinically achievable concentrations | Repositioning of fluoroquinolones | The comprehensive reviews provide evidence that currently commercially available fluoroquinoloes used to treat bacterial infections can be repositioned as anti-cancer agents due to their pro-apoptotic-, anti-proliferative-, and anti-metastatic potential. | 72-75 |
| Ciprofloxacin | Bladder- and colon carcinoma cell lines | Anti-proliferative effects at clinically achievable concentrations | Interaction with topoisomerases may cause an anti-proliferative effect in cancer cells | High urine- and fecal concentrations of ciprofloxacin may translate into clinical efficacy in treatment of bladder- and colon carcinomas | 76, 77 |
| Ciprofloxacin | Hematopoetic-, melanoma-, Glioblastoma-, osteosarcoma-, ovarian-, lung cancer cells | Anti-proliferative effects at supra inhibitory concentrations. Synergism with tumor necrosis factor and interferon | Interaction with topoisomerases may cause an anti-proliferative effect in cancer cells | Anti-proliferative in vitro effects were caused by unphsiologically high concentrations. However, immunomodulatory effects of fluoroquinolones were triggered by high concentrations as well, so that this phenomenon may be due to in vitro methodological effects.  However, meta-analyzes showed that in cancer patients prophylaxed for infections with fluoroquinolones versus patients receiving placebo or no treatment the all-cause mortality was reduced, thus indicating that the doses used for treatment of infectious diseases exert anti-neoplastic activies as well (86-88). | 78-85  86-88 |
| Moxifloxacin  Gatifloxacin  Ciprofloxacin | pancreatic cancer cell-lines. | Inhibitory effects of CPX at 200 + 400mg/L, MXF at 400mg/L, GAT at 400mg/L  Blockade in S-phase of cell cycle. Trigger of the intrinsic mitochondrial apoptotic pathway. Synergism with  cisplatin. GAT induced S- + G2 cell cycle arrest and synergized with cisplatin + gemcitabine | Ciprofloxacin is known to inhibit proliferation of cancer stem cells. Thus, 8-methoxyquinolones were studied. | Anti-proliferative in vitro effects were caused by unphsiologically high concentrations. However, immunomodulatory effects of fluoroquinolones were triggered by high concentrations as well, so that this phenomenon may be due to in vitro methodological effects. | 89, 90 |
| Levofloxacin | Lung cancer cell lines,  xenograft lung tumor model | Inhibition of proliferation and induction of apoptosis.  Inhibition of mitochondrial electron transport chain complex I and III, leading to inhibition of mitochondrial respiration and reduction of ATP production. In addition, levofloxacin significantly increases levels of ROS, mitochondrial superoxide and hydrogen peroxide *in vitro* and oxidative stress markers (HEL and 4-HNE) *in vivo* | Confirmatory study that levofloxacin, too, exerts an anti-cancer effect. | Levofloxacin targets mitochondrial functions. | 91 |
| CIP, OFX, LVX, NOR, FLE, GAT, ENX, PFX, SPX, LOM,  Pipemidic acid,  Oxolinic acid,  Difloxacin,  Balofloacin,  Pazufloxacin,, Nadifloxacin, | Human embryo kidney cells (HEK-293).  Quinolone concentrations 5 to 150μM. | ENX, NOR, FLE, OFX, balofloxacin, prulifoxacin, and pazufloxacin enhanced statistically significantly RNAi activities. The EC_50_ of ENX = 30μM as compared to an IC_50_ for topoisomerase II inhibition = 1,485μM.  Only ENX, NOR, PFX affected micro-RNA activity, thus enhancing DNA repair in mammals. | Quinolones may affect gene functions in mammals (detected by analysis of the RNA interference pathway, RNAi) and may trigger the eukaryotic DNA-damage repair system via micro-RNAs which play a role in e.g. carcinogenesis. Thus, the question was addressed if quinolones may affect genes functions in mammalian cells. | Fluoroquinolones can be used as enhancers of RNAi efficiency and enhancers of DNA repair by augmenting the synthesis of RNA-interference pathway related endoribonucleases DICER.  Concentrations needed are 15-fold lower than those needed to inhibit human topoisomerase II. | 92-94 |
| Enoxacin  Norfloxacin,  Pefloxacin,  Levofloxacin | primary marrow and Raw 264.7 cells.  Quinolones concentrations 1 to 100μM | ENX inhibits binding between the B-subunit of vacuolar H^+^-ATPase (V-ATPase) and microfilaments, and also between osteoclast formation and bone resorption *in vitro*.  PFX, NOR, and LVX had similar inhibitory effects on osteoclasts compared with ENX. While ENX and NOR stiumulated micro-RNA activity PFX and LVX did not. | Same as above | The selective inhibition of osteoclasts by fluoroquinolones may be beneficial in the treatment of osteoporosis and other bone pathologies. | 95-98 |
| Enoxacin | 12 cancer cell lines from seven common malignancies, as well as orthotopic, and metastatic mouse models | Enoxacin enhanced the production of micro-RNAs with tumor suppressor functions by enhancing the binding affinity of TAR RNA-binding protein 2 to pre-micro-RNAs thereby increasing pre-micro-RNA processing by DICER rather than DROSHA. | Fluoroquinolones may modulate synthesis of RNA-interference pathway related endoribonucleases DICER and DROSHA | Enoxacin could be used for restoring the distorted microRNAome of cancer cells to a more physiological setting. | 99, 100 |
| Enoxacin. Ofloxacin | five prostate cancer lines; human melanoma cell line A375 | Enoxacin inhibited growth of five prostate cancer lines and restored global expression of micro-RNAs. Enoxacin inhibited growth and viability of the human melanoma cell line A375 much stronger than ofloxacin by modulating micro-RNAs which process alternative splicing machinery thus activating the tumor suppressor p53 | Same as above | Enoxacin or possibly other fluoroquinolones, too, could potentially be repurposed as activators of p53 tumor suppressor in cancers overexpressing MdmX oncoprotein; p53 activation might contribute to the activity of enoxacin towards human cancer cells. | 101, 102 |
| **Miscellaneous** | | | | | |
| rifampicin | BALB/c-mouse-sarcoma L-1; | 7- to 10 days treatment,  human doses on a mg/kg/day basis | Analysis of the role of immune functions in cancer treatment. In theory, rifampicin may inhibit the mitochondrial DNA-dependnet RNA polymerase | Tumor suppression. Suppression of delayed type hypersensitivity + of spleen lymphocyte proliferation. | 2-3 |
| Fusidic acid | 3 human Burkitt’s lymphoma cell lines, 1 ovarian serous adenocarcinoma, 1embryonic kidney cell line | Cytotoxicity in 3 cell lines only (embryonic kidney cell line, ovarian serous adenocarcinoma, 1 Burkitt’s lymphoma cell line)  EC_50_ 0.145 to 0.181 mM | Inhibition of mitochondrial functions by protein synthesis inhibiting antibiotics may in theory augment the activities of recombinant immunotoxins developed for cancer treatment. | EC_50_ values were within the range of achievable plasma concentrations of approximately 0.058-0.387mM . Fusidic acid showed synergism with recombinant immunotoxins | 46 |
| Clindamycin | H1299cells + 100mg/L clindamycin in conditioned medium | Clindamycin inhibited mitochondrial translation, reduced ATP concentrations by >30%, induced MMP-13 expression almost 2-fold | Verification/falsification of the hypothesis that clindamycin induces senescence-like responses, especially the mitochondria-to-nucleus stress signals and MMP-expression in chloramphenicol-treated H1299 cells. Other mitochondrial translation inhibitors served as comparators. | Clindamycin might trigger a novel mitochondrial stress signal that results in an increase of cancer cell invasion and an acceleration of cancer progression.  Chloramphenicol and tetracyclines caused analogous effects. | 44, 45 |
| Metronidazole | MCF-7 (estrogen receptor positive) and MDA-MB-231 (estrogen receptor negative) breast cancer cell lines | Metronidazole (250mg/L) increased growth of MCF-7 cell lines after 24h of incubation, but it reduces cell viability in concentrations 1 and 10 mg/L after 72h incubation. Significant increase of MDA-MB-231 cell viability was recorded after incubation with 250mg/L for 24h and 72h | Evaluation of metronidazole’s cytotoxicity and its effect on DNA synthesis in breast cancer cell lines | Metronidazole exerted different dose- and time- dependent effects on human breast cancer cell lines characterized by presence or absence of estrogen receptors. These discrepancies may probably be affected by estrogen signalling. | 103 |
| Nitrofurantoin | 3 human bladder cancer cell lines exposed to up to 2,000mg/L for 96h | Significant cytotoxicity in the three cell lines at 7.8mg/L | Evaluation of the cytotoxicity of commonly used urinary antibiotics at clinically relevant concentrations | Administration of antibiotics after resection of bladder tumors might prevent seeding of cancer cells thus decreasing the recurrence rate | 1 |
| Nitroxoline | Human umbilical vein endothelial cells (HUVEC), The human bladder cancer cell line, KU7.  Breast cancer HCC1954 cells were implanted inot BALB/c, nu/nu-NCr mice | Nitroxoline IC_50_-values: MetAP2 activity in = 54.8 nM,  SIRT1= 20.2 μM,  HUVEC proliferation = 1.9 μM.  Mice (five per group) treated with nitroxoline showed a 60% reduction in tumor volume in breast cancerxenografts | Angiogenesis has an important role in tumor growth and metastasis. The type 2 methionine aminopeptidase (MetAP2) protein is a potential anti-angiogenic target. If had been shown that clioquinol is a proteasome inhibitor. Nitroxoline is a clioquinol analog. Furthermore, the silent information regulator 2 family of nicotinamide adenine dinucleotide–dependent deacetylases (sirtuins [SIRTs]) play important roles in gene silencing and DNA repair. Nitroxoline shows structural similarities to sirtuins , so that SIRT may thus be another potential target. | Nitroxoline is a novel inhibitor of angiogenesis and has a dual mechanism of action inhibiting both MetAP2 and SIRT1. The concurrent inhibition of MetAP2 and SIRT1 is synergistic. | 104 |
| Nitroxoline | 11 human cancer cell lines.  mouse models of orthotopic bladder cancer. Oral treatment with 30mg/kg b.i.d. to 240 mg/kg b.i.d. for 21 days | Minimal and maximal IC_50_-values ranged from  1.68 to 20.82 μM against 2 different bladder transitional carcinoma cell lines.  Dose dependent inhibition of tumor growth in the mouse model ranging from 46.4% to 73.6% | The experiments were designed referring to the study mentioned above(90) | Nitroxoline exhibited concentration- or dose‑dependent anti‑cancer activities in vitro and in urological tumor orthotopic mouse models. | 105 |
| Nitroxoline | Viability and proliferation of human cholangio-carcinoma cell lines, HuCCT1 and Huh28.  Quantification of FoxM1 signaling via expression levels of the target genes were determined | IC_50_ against  HuCCT1=3.69μM and Huh28= 4.49μM.  Decreased FoxM1 expression concomitant with downregulation of various FoxM1’s down-stream targets including cdc25b, CENP-B, and survivin. In addition, expression of matrix metalloproteinase (MMP)-2 and MMP-9 was inhibited | Fork head box M1 (FoxM1) is an oncogenic transcription factor. expression and transcriptional activity of FoxM1 could be inhibited by several proteasome inhibitors like clioquinol with which nitroxoline shows structural similarities. | Nitroxoline disrupted FoxM1 signaling and interfered with the zinc-coordinated active site of the enzymes, suggesting that nitroxoline inhibited directly MMP function. | 106 |
| Nitroxoline | U87 and U251 glioma cell lines,  mouse glioma model,  small-cell lung cancer (SCLC) cells | Nitroxoline inhibited the proliferation and invasion of glioblastoma cells in a time- and dose-dependent manner in vitro. Growth inhibition was associated with cell-cycle arrest in G1/G0phase and induction of apoptosis via caspase 3 and cleaved poly(-ADP-ribose) polymerase | The experiments were designed referring to the study mentioned above(90) |  | 107 |
| Nitroxoline | Small cell lung cancer cell line | Induction of apoptosis by suppressing antiapoptotic proteins (such as Bcl-2 and MCL1) andupregulating proapoptotic protein Bim.  MDM2 expression was downregulated by inducing its proteasomal degradation, and thus expression of p53, which was a substrate protein of MDM2, was upregulated | The experiments were designed referring to the study mentioned above(90) | Nitroxoline displayed anti-small cell lung cancer activity by suppressing MDM2 expression, which suggested that nitroxoline may have the potential for treatment of Small cell lung cancer by targeting the MDM2/p53 axis | 108 |
| Bedaquiline | MCF7 breast cancer cells | In MCF7 breast cancer cells mitochondrial oxygen‐consumption and glycolysis was inhibited and the propagation and expansion of MCF7‐derived CSCs was blocked with an IC_50_=1μM.  bedaquiline targeted preferentially cancer stem cells but not normal fibroblasts and marginally mature cancer cells. | Bedaquiline is a diaryl‐quinoline compound, that inhibits bacterial ATP‐synthase. Therefore, bedaquiline might also target the mitochondrial ATP‐synthase thus exerting anti-cancer activities. | Bedaquiline inhibited cancer stem cells leaving mature cancer- and normal cells intact. mechanism(s) of selectivity are unknown at present. Furthermore, the authors showed structural similarities between bedaquiline and trans-piceatannol as well as trans-resveratrol which are known natural flavonoid inhibitors of the mitochondrial ATP‐synthase and show anti‐aging properties and are also active as antibacterials. | 109 |
| Bedaquiline | Various human lung cancer cell lines.  Xenograft lung cancer mouse model | Inhibition of proliferation and induction of aptosis in lung cancer cell lines due to inhibition of mitochondrial respiration and glycolysis, resulting in ATP reduction and oxidative stress. These results were confirmed in vivo in the xenograft lung cancer mouse model | The experiments were designed referring to the study mentioned above (95) | Energy depletion resulted in inhibited lung tumor cell progression and in angiogenesis. | 110 |

Table S2
Interaction of antibiotics with metallo-matrixproteinases (MMPs; TNFα = tumor-necrosis factor α; CXCL8 = C-X-C motif chemokine ligand 8 (interleukin 8); NF-kB = nuclear factor-kB; PMA, phorbol 12-myristate 13-acetate; PBMCs, peripheral blood mononuclear cells; NE = no measurable effect; IC_50_= concentration inhibiting enzyme activity to 50%; ERY=erythromycin; CLR=clarithromycin; AZM=azithromycin; TEL=telithromycin; SOL=solithromycin; OFX = ofloxacin; LVX = levofloxacin; CIP = ciprofloxacin; MXF = moxifloxacin; NOR =norfloxacin; DOX = doxycycline; MIN = minocycline; RIF = Rifampicin; INH = Isoniazid; PAS = para-Aminosalicyclic acid; n.d. = not done; NE = no effect

| Antibiotic | Model, target | MMPs and/or cytokines inhibited | Effect, comment | Ref |
| --- | --- | --- | --- | --- |
| **ß-lactams** | | | | |
| Cephalothin | *Patients* with loose hip prosthesis requiring revision vs. non-inflammatory knee synovial tissue sampled by arthoskopy re. meniscus injury | Significant decrease of MMP activity by 31.9% at 100mg/ml cephalothin, and 13.6% at 1,000mg/ml. Doxycycline, tetracycline, and gentamicin served as comparators. Only tetracycline inhibited MMP by 45.2% at 500mg/L, whereas doxycycline and gentamicin were ineffective. | cephalothin can inhibit MMP activity in reactive periprosthetic tissue and thus reduc the tissue destruction.  These findings were questioned (112) because of methodological reasons and as cephalothin is not a chelator. However, from the two chelating tetracyclines used as comparators, only one inhibited MMP-activity and ß-lactams are known to contain electron donor groups binding metal ions. | 111, 112 |
| Amoxicillin plus  Metronidazole | *Patients* with generalized aggressive periodontitis were treated with 500mg orally t.i.d. each for 7days versus scaling and root planning alone. Sampling at baseline and 3 + 6 months later | Total MMP-1 levels were significantly decreased in both groups (P < 0.05) at 3 and 6 months as compared to baseline. However, differences among groups at 3 or 6 months were not different. Patients with deeper pockets benefitted from antibiotic treatment with a more stable healing compared to the shallow and moderately deep pockets.  TIMP-1 concentration levels increased in both groups at 3 and 6 months while the difference did not reach statistical significance. | There was a trend towards better improvement in the verum group as compared to the placebo group in the subset of patients with deep pockets. | 113 |
| **Macrolides** | | | | |
| Erythromycin  Clarithromycin,  Azithromycin,  Telithromycin,  Solithromycin, | Effects of macrolides on release of proinflammatory mediators, such as MMP9, TNFα, CXCL8, in macrophage like PMA differentiated U937 cells and PMBCs exposed to 10, 33, 100μM | \| Inhibitory effects of macrolides \| \| \| \| \| \| \| --- \| --- \| --- \| --- \| --- \| --- \| \|  \| IC_50_ (μM) \| \| \| \| \| \|  \| ERY \| CLR \| AZM \| TEL \| SOL \| \| CXCL8 \| NE \| 506 \| NE \| NE \| 78.2 \| \| TNFα \| NE \| 422 \| NE \| NE \| 41.6 \| \| MMP9 \| NE \| 118 \| 212 \| 83.4 \| 14.9 \| | Macrolides exerted differential effects on the producrion of pro-inflammatrory cytokines and the synthesis of MMP9. Solithromycin was the most effective macrolide under these experimental conditions. The effect of solithromycin on MMP9 production was confirmed in smoke exposed mice.  These effects including inhibition of MMP9 may likely be due to the fact that macrolides inhibit transcription of NF-kB regulated expression of e.g. TNFα, CXCL8 and MMP9. | 114 |
| Roxithromycin,  Josamycin | Expression of MMP9 in LPS stimulated neutrophils or nasal fibroblasts and of MMP2 and -9 in TNF-α stimulated nasal polyp fibroblasts | Roxithronycin inhibited MMP2 and -9 expression and suppressed NF-kB activation at >5mg/L via inhibition of NF-κB and activator protein-1 activation.  Josamycin had no effect. | Roxithromycin inhibited the activation of NF-kB and activator protein-1 induced by TNF-α and resulted in suppression of MMP production. | 115, 116 |
| Clarithromycin | murine cardiac allograft recipients | Suppression of MMP-9 expression in the allografts was associated with  suppression of smooth muscle cell migration and proliferation. | Clarithromycin may be useful in the prevention of acute and chronic rejection through the inhibition of  MMP-9 activity.. | 117- 119 |
| Clarithromycin | *Patients* with severe refractory neutrophilic asthma received either 500mg b.i.d. clarithromycin or placebo for 8 weeks. | 8 week clarithromycin treatment reduced sputum CXCL8, neutrophils, neutrophil elastase, and MMP9 concentrations and also improved quality of life scores in patients with refractory neutrophilic asthma. | Clarithromycin therapy modulated pro-inflammatory cytokine concentrations and neutrophil accumulation and activation in the airways of patients with refractory asthma. | 120 |
| Azithromycin | Human bronchial epithelial cells exposed to 30 mg/L for 6, 24, or 48h.  Analysis of global gene expression using RNA microarrays | Exposure to azithromycin for 6h prevented the upregulation of MMP1, -2, -9, -10, -13, -14, -28 genes, and CXCL1, -2, -3, -5, -6, -14, -16, CLCR7 which were triggered by inflammatory stimuli. Incubation for 24h resulted in a downregulation of MMP1, -9, -10, -13 genes. | Alterations of inflammatory responses by clinically achievable azithromycin concentrations were highly variable and gene-specific, with an overall tendency toward global down-regulation. The striking down-regulation of the RNA of e.g. MMP9 gene is likely to be highly relevant to clinical outcomes. | 121 |
| Azithromycin | Human bronchial epithelial- and MRC-5 cells exposed to 5, 10, 30mg/L for 72h. Measurements of MMP concentrations. | Respiratory epithelial cell-derived MMP-3 was suppressed concentration dependently; maximal suppression (60%) was observed using 30mg/L azithromycin. In contrast, MMP-1 and -3 secretion from MRC-5 fibroblasts were increased. | These data demonstrate that the effects of azithromycin are cell specific | 122 |
| Azithromycin | primary bronchial epithelial cells,  airway smooth muscle cells,  Exposition to 5, 10, 20mg/L for 48h.  Monocytic THP1 cells exposed to 1, 5, 10, 50, 100, 500μM azithromycin or minocycline. | Azithromycin inhibited MMP-2 and MMP-9 release from primary bronchial epithelial cells and airway smooth muscle cells.  Concentrations of IL-8, GM-CSF were reduced as well.  Azithromycin but not minocycline reduced MMP-9 mRNA and protein levels without affecting NF-κB in endotoxin-challenged THP-1cells. Azithromycin had no effect on MMP9 activity while high minocycline conentrations inhibited enzyme activity. | Azithromycin decreased concentrations of MMP-9 by reduction of gene- and protein expression | 123- 125 |
| Azithromycin | Topical ocular treatment with azithromycin (15 mg/g b.i.d.) or vehicle. After three days lipopolysaccharide was injected subconjunctivally | Gene expression for Il-6 and TNF-α was almost unchanged and was increased for cyclooxygenase-2. Gene expression for MMP-2 and -9 was reduced 2.3- and 1.6-fold. | Azithromycin ameliorates inflammation in a rat model of acute conjunctivitis. | 126 |
| Azithromycin | Matrix remodelling in *stable patients* and in *lung transplant patients* suffering from neutrophilic type of chronic lung allograft dysfunction/neutrophilic reversible allograft dysfunction (nCLAD/NRAD). MMP2 and -9 were measured via ELISA before and after 3 to 6 months of azithromycin therapy (dose not specified). | The nCLAD/NRAD patients showed higher airway neutrophilia and MMP-9 concentrations compared to the control group.  Although azithromycin significantly reduced MMP2 and -9 concentrations in transplant patients, the levels remained higher compared to control patients in whom MMP concentrations in BAL were below the limit of detectability. | Treatment of lung transplant patients with azithromycin did not completely restore chronic inflammation in the airways. The study also suggested that preventive therapy may yield added value to curative therapy. | 127 |
| **Chloramphenicol** | | | | |
| Chloramphenicol | H1299cells + 10-100mg/L chloramphenicol in conditioned medium | Chloramphenicol induced  MMP13 expression and increase MMP-13 protein in conditioned medium,  resulting in an increase in cancer cell invasion.  Chloramphenicol also activated c-Jun N-terminal kinases  and phosphatidylinositol 3-kinase/Akt signaling, leading to c-Jun protein phosphorylation. The activated c-Jun protein has been proven to activate binding to the MMP-13 promoter and also  upregulate the amount of MMP-13. | Chloramphenicol can accelerate cancer progression | 44 |
| **Oxazolidinones** | | | | |
| Linezolid | Pneumonia model in BALB/c mice inoculated with methicillin-resistant S. aureus Comparison of the effects of linezolid (80mg/kg IV b.i.d.) and vancomycin (110 mg/kg IV b.i.d.) | MMP9 concentrations did not differ between the treatment groups. | Linezolid exerted no significant effect on MMP9 expression under these experimental conditions. | 128, 129 |
| **Tetracyclines** | | | | |
| Tetracycline | In vitro | 500mg of tetracycline per ml inhibited MMP activity (45.2%) |  | 111 |
| Doxycycline | Various in vitro studies  osteosarcoma-, prostate-, breast cancer-, melanoma cell lines | IC_50_ of doxycycline for various MMPs:  MMP1 = >200μM  MMP8 = 1-10μM  MMP9 = 1-48-(608*) μM  MMP13 = 5-30μM  Induction of apoptosis, decrease of invasion and metastatic potential | Doxycycline may exert beneficial effects in various types of cancer. A potentiation of its activity was noted in combination with a peptidomimetic MMP inhibitors (batimast, marimast) | 130-140  141* |
| Doxycycline | Review of clinical studies.  Treatment of *patients* with periodontal disease with sub-antimicrobial dose of 20 mg b.i.d., compared to “regular or antibiotic-dose” 100 mg q.d. or b.i.d.) | Suppression of MMP9 in the periodontal lesion thereby inhibiting the pathologic degradation of various collagens, including types I, III, and IV, while preserving other constituents of the periodontal tissues (fibronectin, proteoglycan groundsubstance, elastin and basement membranes) and inhibiting bone resorption. | The first of two approvals for “non-antimicrobial” or “host-modulating” antibiotics by regulatory authorities in North America and Europe as adjunctive treatment for the management of chronic periodontitis | 142-146 |
| Doxycycline | Treatment of *patients* with rosacea with a subantimicrobial dose of 40mg b.i.d. | Review of clinical studies: Suppression of MMPs 1, -2, -9, -12, -13 and dowregulation of TNFα and Il-1ß. | Marketing authorizations granted for treatment of rosacea with sub-antimicrobial dose  of 40 mg b.i.d. .  Minocycline 100mg was equally effective but antibacterially active, so that resistance could emerge | 147, 148 |
| Doxycycline | Ophthalmic disease  *Patients* | Review of clinical studies: Reduced expression of MMP2, -9 and of TNFα | Oral doxycycline may provide an effective treatment of ocular diseases. However, prospective, randomized clinical studies are desirable. | 149, 150 |
| Doxycycline | Human corneal epithelial cells | Suppression of stimulated MMP1, -10, and -13at both, the mRNA- and protein levels | Doxycycline may be useful for treatment of ocular surface diseases | 151 |
| Doxycycline | Ocular surface repair.  Epithelial cells and keratocytes exposed to 1, 20, 100, 200 and 1,000μM | \| DOX  μM \| MMP2 activity \| MMP2 synth. \| MMP9  synth. \| MMP1  synth. \| \| --- \| --- \| --- \| --- \| --- \| \| Epithelial cells (%inhibition vs. controls) \| \| \| \| \| \| 100 \| 34 \| 5 \| 5 \| 6 \| \| 200 \| n.d. \| 14 \| 23 \| 30 \| \| 1,000 \| 81 \| n.d. \| n.d. \| n.d. \| \| Keratocytes (%inhibition vs. controls) \| \| \| \| \| \| 100 \| n.d. \| 17 \| NE \| 9 \| \| 200 \| n.d. \| n.d. \| NE \| n.d. \| | The concentrations needed to achieve moderate inhibitions of MMP1, -2, -9 synthesis are relatively high and may exceed achievable concetrations in tears upon oral administration. However, clinical reports indicate that oral doxycycline is effective in the treatment of ocular infections. | 152 |
| Doxycycline | Cardiovascular diseases.  Reviews of various in vitro and in vivo models. | IC_50_ of doxycycline for  MMP1=452μM  MMP2=56μM  MMP3=32μM  MMP9=30μM  In some models minocycline inhibited MMP3 only with an IC_50_=290μM; it was a more potent inhibitor of MMP9 in other models. | Attenuation of MMP2 and -9 are  involved in hypertension- and myocardial infarction induced cardiovascular remodeling.  Their attenuation but not total inhibition may be a good strategy to ameliorate the sequelae.  Results generated varied probably model dependently. | 153-  155 |
| Doxycycline | Human aortic smooth muscle- and aortic aneurysm tissue explants.  *Patients*, elective open aneurysmal repair.  Patients were treated with 0, 50, 100, or 300mg/day for 2 weeks. | IC_50_ of doxycycline for MMP2 expression=6.5mg/L in both models.  Independently of its dose expression of MMP8 + -9 was statistically significantly reduced, and expression of MMP12 tended to be reduced.  Aortic wall neutrophils were selectively reduced by 72% and the aortic wall cytotoxic T-cell content was reduced by 95%. | Doxycycline treatment resulted in a profound but selective suppression of aortic wall inflammation | 156, 157 |
| Doxycycline | *Patients* with small abdominal aortic aneurysm to reduce expansion of aneurysm. Treatment with 150mg/day for 3 months vs. placebo. (158)  *Patients* undergoing endovascular aneurysm repair (EVAR).  DOX (100mg b.i.d.) or placebo for 6 months following EVAR (159). | The aneurysm expansion rate in the DOX group was significantly lower than that in the placebo group during the 18 months follow up period (158).  Plasma MMP-9 decreased below baseline in DOX treated patients while there was an insignificant increase in the placebo group. In patients with endoleaks at 6 months, plasma MMP-9 increased in 83% of the placebo group, but in only 14% of DOX treated group (159). | Doxycycline retarded expansion of abdominal aortic aneurysm. MMPs were not quantitated in this study (158).  Ongoing aortic degradation after endografting due to persistent MMP release can be inhibited by doxycycline therapy (159). | 158, 159 |
| Doxycycline | *Patients* with anabdominal aortic aneurysma were treated 100 mg orally twice a day for 7 days before surgery | treatment was associated witha slight 24.4% reduction of MMP2 and 55% reduction of MMP9 concentrations. MMP9 mRNAwas reduced by 82%. In cultured THP-1 monocytes stimulated with phorbol ester, the expression of MMP-9 protein and mRNA were both decreased | DOX may influence connective tissue degradation within human aneurysm tissue by reducing monocyte/ macrophage expression of MMP9 mRNA and by suppressing the post-trans-lational activation of proMMP2. Treatment with DOX may be a particularly effective strategy for achieving MMP inhibition in patients with an anabdominal aortic aneurysma | 160 |
| Doxycycline | Stable COPD *patients* treated with 100mg doxycycline qd or b.i.d. as per Body Mass Index as an add on therapy. | Concentrations of MMP-2 and MMP-9/ TIMP-1-ratio were decreased significantly. Concentrations of inflammatory cytokines were also reduced. | Improvement in lung function and quality of life in COPD patients may probably be attributed to the reduced MMP concentrations as well as anti-oxidant- and anti-inflammatory activity of doxycycline. | 161 |
| Doxycycline | Stable COPD *patients* were treated with 100mg doxycycline for 3 weeks as an add on therapy.  In order to exclude inflammation by bacterial colonisation patients must have had negative sputum cultures prior to enrolment. | MMP8 and -9, IL-6and IL-8 concentrations as well as lung function parameters were not affected by doxycycline.  Systemic inflammation by means of CRP was also not influenced by doxycycline. | Doxycycline did neither affect neutrophil content nor the amount of MMP8 and -9 in sputum. This is in contrast with earlier clinical studies with doxycycline in periodontitis- (141-145), rosacea- (146, 147) and aneurysma patients (155), and the exploratory study in COPD patients (156).  Differing expression of MMP-8 and -9 can be explained by the low cell count as well as a low percentage of neutrophils in the induced sputum at the beginning of this study compared to previous trials. This might be due to the absence of bacterial colonization in this patient group. | 162 |
| Doxycycline | Tuberculosis  Review, various in vitro and in vivo models.  *Patients* with/without HIV coinfection | Doxycycline suppressed TB-dependent expression of MMP1 and -9 in preclinical models.  MMP concentrations in induced sputum of patients with HIV were decreased. | TB patients may benefit from adjunctive doxycycline administration. | 163, 164 |
| Doxycycline | Cystic fibrosis *patients*, 100mg doxycycline b.i.d. for 8 days | Doxycycline reduced total sputum MMP-9 concentrations by 63.2% and active MMP-9 concentrations by 56.5%; TIMP-1 sputum concentrations increased 1.6-fold. | Doxycycline reduced dysregulated MMP-9 concentrations in sputum, so that CF-patients may benefit from adjunctive doxycycline treatment. | 165 |
| Doxycycline | Reviews on the role of doxycycline in  wound healing.  In vitro studies, animal models, clinical trials. | In general, doxycycline enhances healing of chronic wounds. | Doxycycline may be a treatment option in managing chronic wounds. | 166-171 |
| Doxycycline | *Patients* with non-healing venous leg ulcers  Tretament with either 20mg or 100mg b.i.d. | In the high-dose doxycycline group, the reduction in median ulcer area was 48% and there was a significant reduction in wound fluid total MMP1; no effects were observed in the low dose group. Important to note: median wound fluid DOX-concentrations after 4 weeks of treatment were 0.2 and 2.3mg/L in the low- and high-dose groups, respectively, which are lower than concentrations inhibitimg MMPs in vitro. | Doxycycline 100 mg twice daily may improve the healing rate of recalcitrant leg ulcers | 172, 173 |
| Doxycycline | Model of acute lung injury in mongrel dogs. Treatment with 30mg/kg or 60mg/kg vs. control. | Plasma concentrations of MMP9 were decreased significantly in the high dose group. | Doxycycline reduced acute lung injury induced by cardiopulmonary bypass. | 174 |
| Tigecycline | Rat model of burn wound infections by *S. aureus.*  Immunochemical analysis of MMP9. | Infected rats treated with tigecycline showed a significant decrease in MMP-9 expression both in epithelium and in dermis compared with rats treated with teicoplanin.  Number of cells in % of total cells examined in epithelium and dermis, respectively, expressing MMP9:  20% and 50% | Tigecycline modulated MMP9 expression, thus supporting treatment of burn injuries. | 175 |
| Doxycycline  Minocycline | Various | Inhibition of MMP2 and -9 expression | The potential use of teracyclines in treatment of cerebral diseases or tumor treatment is discussed | 176-187 |
| **Fluoroquinolones** | | | | |
| Norfloxacin,  Ofloxacin,  Ciprofloxacin,  Nalidixic acid | Human tendon derived cells | NOR, OFX, CIP reduced MMP13 mRNA  NOR, CIP increased MMP1 mRNA expression; OFX had no effect on MMP1. Nal reduced expression minimally, if at all. | The fluoroquinolones tested exhibited contrasting effects because fo unknown reasons.  In general, studies on the potential mechaninsms of fluoroquinolone induced tendinopathy correlate this adverse reaction with an increased expression of MMPs | 188 |
| Ciprofloxacin | Human dermal fibroblast cultures exposed to 20, 50, 100mg/L CIP | Ciprofloxacin induced extracellular signal-regulated kinase (Erk)1/2 phosphorylation, and Erk1/2 blockade completely prevented MMP1 upregulation. | CPX may be an attractive therapy for systemic sclerosis, skin and lung fibrosis.  A recent clinical study demonstrated that oral administration of CPX (250mg b.i.d., versus placebo) for 6 months reduces the severity of symptoms affecting the skin of patients with systemic scleroderma (189) | 189  190 |
| Ofloxacin,  Levofloxacin,  Ciprofloxacin | Corneal epithelium.  Tear drops OFX 0.3%, LVX 0.5%, CIP 0.3% | Expression of MMP1, -2, -8, and -9 were upregulated as compared to artificial tear fluid group. | MMPs were upregulated as a result of wound healing process | 191, 192 |
| Moxifloxacin | Primary normal human bronchial epithelial cells | Expression of respiratory epithelial cell-derived MMP-1 and -9 was suppressed. Maximal suppression was achieved with 20mg/L. In contrast, MXF increased MMP-1 and -3 secretion from MRC-5 fibroblasts. | The effects of moxifloxacin are cell specific. | 122 |
| Moxifloxacin | Primary gingival fibroblasts were infected with *Porphyromonas gingivalis* or with *Aggregatibacter actinomycetemcomitans* | Moxifloxacin exerted a good antibacterial activity but it did not modulate MMP3 release. | Moxifloxacin could be selected in refractory cases of periodontitis, as an alternative to conventional antibiotic therapy. | 193 |
| **Miscellaneous** | | | | |
| “Antibiotics” | Antibiotic treatment of cystic fibrosis *patients* with pulmonary exacerbations. “Antibiotic” treatment was not specified | Antibiotic treatment of cystic fibrosis patients with pulmonary exacerbations led to a decrease of MMP1, MMP8 and MMP9 protein concentration. | In general, antibiotic treatment reduced concentrations of MMPs in patients. However, specific conclusions can not be drawn as treatment regimens were not specified | 194 |
| Rifampicin,  Isoniazid | Human bronchial epithelial- and MRC-5 cells exposed to 5, 10, 30mg/L for 72h. Measurements of MMP concentrations. | Rifampicin downregulated epithelial cell-derived MMP3 in a dose-dependent manner. MMP3 gene expression was suppressed to below baseline. However, MMP1 and -9 mRNA accumulation and secretion were not altered by pretreatment with rifampin, nor was MMP-3 secretion altered significantly.  Isoniazid was used as a comparator; it did not affect any MMP. | Suppression of epithelial MMP3 production by rifampicin may be a consequence of rifampicin’s actions on the prostaglandin pathway. | 122 |
| Rifampicin,  Isoniazid | TDM granuloma- and Mtb/Matrigel granuloma mouse model; mice were infected intranasally. INH (12.5mg/kg/day) and RIF (5mg/kg/day) were added to the drinking water starting 14 days post infection. MMP inhibitors were injected intraperitoneally every second day starting 7 days post infection. | An increased delivery and/or retention of rifampicin and isoniazid in infected lungs was observed resulting in enhanced drug efficacy. This effect is likely due to inhibition of MMP2 and -9 activity leading to an increase in pericyte-covered blood vessel numbers and appears to stabilize the integrity of the infected lung tissue.  Marimastat, batimastat, and Sb-3ct increased drug efficacy significantly, whereas the effect of prinomastat was not statistically significant. | MMP inhibition enhanced efficacy of anti-mycobacterial agents via improved drug delivery.  These data demonstrate that antibacterial efficacy and MMP inhibition are closely but indirectly interrelated. | 195 |
| para-Aminosalicyclic acid | Human monocyte-derived macrophages | PAS blocked prostaglandin expression thus inhibiting indirectly *M. tuberculosis*-driven MMP1 but not -7 gene expression and secretion. PAS acted by blocking PGE2 production without affecting *M. tuberculosis* growth. | Data suggest that PAS decreased MMP1 activity by inhibiting the p38 mitogen-activated protein kinase-prostaglandin (MAPK-PG) signaling cascade.  MMPs are produced in response to mycobacterial infections. p38 MAPK is phosphorylated in macrophages surrounding granulomas. p38 upregulates MMP activity and owregulates TIMP secretion. Prostaglandin is a key downstream effector of p38 activity. | 196 |
| Daptomycin | *Patients* with MRSA infected diabetic foot syndrome.  Tretament with 4-6mg/kg/day for maximally 14 days. | MMP9 decreased by 25% on day 7 and almost to baseline at day 21 paralleled by an increase in TIMP-1. Furthermore, local IL-6 concentrations decreased within the first 3 days. | In addition to a reduction of wound size several beneficial processes at the molecular level were recorded. | 197 |
| Dalbavancin,  Vancomycin | Mouse model of MRSA skin infection.  Daily treatment with vancomycin (10mg/kg) and weekly with dalbavancin, at day 1 (20 mg/kg) and day 8 (10 mg/kg) | MMP1 and MMP9 were decreased in uninfected and in both treatment groups as compared to untreated animals. | Decreased MMP levels contribute to the anti-infective activity of both agents. | 198 |
| Teicoplanin | Rat model of burn wound infections by *S. aureus.*  Analysis of MMP9 only. | Number of cells in % of total cells examined in epithelium and dermis, respectively, expressing MMP9:  30% and 65% | Teicoplanin modulated MMP9 expression moderately, thus supporting treatment of burn injuries | 175 |
| Bacitracin | U87-MG glioma cells were exposed to 1.25, 2.5, 3.75, and 5.0 mM bacitracin | The expression of MMP2 and phosphorylated focal adhesion kinase (p-FAK) was decreased concentration dependently and the migration ability was reduced. | Bacitracin inhibits protein disulfide isomerase (PDI) via binding with reduced PDI on the cell surface. PDI is associated with tumor cell migration and invasion, so that bacitracin may have the potential to be used as an anti-invasive agent.  Inhibited expression of MMP2 may be a consequence of p-FAK inhibition as FAK is involved in MMP2 production. | 199 |
| Nitroxoline | Human cholangiocarcinoma cell line HuCCT1. Exposition to 10, 20 and 40 μM nitroxoline. | Exposition of HuCCT1 to reduced MMP2 and -9 expression. Furthermore, nitroxoline directly interacted with the active sites of MMP2 and -9 | The activity of nitroxoline resembles that of thiostrepton, a known FoxM1 inhibitor. Therefore it is suggested that nitroxoline may target FoxM1 signaling. Fork head box M1 (FoxM1) is an oncogenic transcription factor frequently elevated in numerous cancers, including cholangiocarcinoma. | 200, 201 |

References

1. Kamat AM, Lamm DL. Antitumor activity of common antibiotics against superficial bladder cancer. Urology 2004; 63: 457-460. [https://doi.org/10.1016/j.urology.2003.10.038](https://doi.org/10.1016/j.urology.2003.10.038)
2. Bannerjee A, Dahiya M, Kumar S. Inhibition of Proliferation of Cervical and Leukemic Cancer Cells by Penicillin G. Asian Pacific J Cancer Prev 2013; 14: 2127-2130. https*://*doi.org*/*10.7314*/*APJCP*.*2013.14.3.2127

# Roszkowski K, Beuth J, Ko HL, Roszkowski W, Jeljaszewicz J, Pulverer G. Influence of 12 Antibiotics on Antitumor Immunity in BALB/c-Mice. Zentralbl Bakteriol Mikrobiol Hyg A 1992; 276: 280-287. [https://doi.org/10.1016/S0934-8840(11)80015-0](https://doi.org/10.1016/S0934-8840(11)80015-0)

# Roszkowski K, Ko HL, van der Waaj D, Roszkowski W, Jeljaszewicz J, Pulverer G. Antibiotic treatment, intestinal aerobic microflora and experimental sarcoma L-1 growth in Balb/c-mice. Zentralbl Bakteriol Mikrobiol Hyg A 1987; 265: 378-3884. DOI: [10.1016/s0176-6724(87)80256-0](https://doi.org/10.1016/s0176-6724(87)80256-0)

1. Roszkowski K, Ko HL, Roskowski W, Jeijesawicz J, Pulverer G. Effects of cefotaxime, clindamycin, mezlocillin, and piperacillin on mouse sarcoma L-1 tumor. Cancer Immunol Immunther 1984; 18: 164-168. DOI: [10.1007/bf00205506](https://doi.org/10.1007/bf00205506)
2. Pochini L, Galluccio M, Scumaci D, Giangregorio N, Tonazzi A, Palmieri F, Indiveri C. Interaction of β-lactam antibiotics with the mitochondrial carnitine/acylcarnitine transporter. Chem-Biol Interact 2008; 173: 187-194. [https://doi.org/10.1016/j.cbi.2008.03.003](https://doi.org/10.1016/j.cbi.2008.03.003)
3. Melone MAB, Valentino A, Margarucci S, Galderisi U, Giordano A, Peluso G. The carnitine system and cancer metabolic plasticity. Cell Death Dis 2018; 9: 228. DOI:10.1038/s41419-018-0313-7

# Anonymos. Ciprofloxacin compared with cephalexin in treating patients with bladder cancer. ClinicalTrials.gov Identifier: NCT00003824. Last updated March 09, 2012. Visited January 15, 2020

1. Li X, Li H, Li S, Zhu F, Kim DJ, Xie H, Li Y, Nadas J, Oi N, Zykova TA, Yu DH, Lee MH, Kim MO, Wang L, Ma W, Lubet RA, Bode AM, Dong Z, Dong Z. Ceftriaxone, an FDA-approved cephalosporin antibiotic, suppresses lung cancer growth by targeting Aurora B. Carcinogenesis 2012; 33: 2548-2557. DOI: 10.1093/carcin/bgs283
2. Ci X, Song Y, Zeng F, Zhang X, Li H, Wang X, Cui J, Deng X. Ceftiofur impairs pro-inflammatory cytokine secretion through the inhibition of the activation of NF-kappaB and MAPK. Biochem Biophys Res Commun 2008; 372: 73–77. [https://doi.org/10.1016/j.bbrc.2008.04.170](https://doi.org/10.1016/j.bbrc.2008.04.170)

# Codini M, Cataldi S, Ambesi-Impiombato F, Lazzarini A, Floridi A, Lazzarini, R., Floridi A, Lazzarini R, Curcio F, Beccari T, Albi, E. Gentamicin arrests cancer cell growth: the intriguing involvement of nuclear sphingomyelin metabolism. Int J Mol Sci 2015; 16: 2307-2319. doi: [10.3390/ijms16022307](https://dx.doi.org/10.3390%2Fijms16022307)

1. Abe J, Yamada Y, Harashima H. Validation of a strategy for cancer therapy: delivering aminoglycoside drugs to mitochondria in HeLa cells. J Pharm Sci 2016; 105: 734-740. [https://doi.org/10.1002/jps.24686](https://doi.org/10.1002/jps.24686)
2. Bellais S, Le Goff C, Dagoneau N, Munnich A, Cormier-Daire V. In vitro readthrough of termination codons by gentamycin in the Stüve–Wiedemann Syndrome. Eur J Hum Genet 2010; 18: 130-132. DOI: [10.1038/ejhg.2009.122](https://doi.org/10.1038/ejhg.2009.122)
3. Lokhande JM, Pawar, S. In-vitro study to evaluate various drugs for potential effects on different cancer cell lines. International Journal of Scientific Research 2018; 7: 419-423. ISSN No 2277 - 8179
4. Zilberberg A, Lahav L, Rosin-Arbesfeld R. Restoration of APC gene function in colorectal cancer cells by aminoglycoside-and macrolide-induced read-through of premature termination codons. Gut 2010; 59: 496-507. [http://dx.doi.org/10.1136/gut.2008.169805](http://dx.doi.org/10.1136/gut.2008.169805)
5. Green L, Goff SP. Translational readthrough-promoting drugs enhance pseudoknot-mediated suppression of the stop codon at the Moloney murine leukemia virus gag–pol junction. J Gen Virol 2015; 96: 3411-3421. <https://doi.org/10.1099/jgv.0.000284>
6. Floquet C, Deforges J, Rousset JP, Bidou L. Rescue of non-sense mutated p53 tumor suppressor gene by aminoglycosides. Nucleic Acids Res 2010; 39: 3350-3362. <https://doi.org/10.1093/nar/gkq1277>
7. Zhang M, Heldin A, Palomar-Siles M, Öhlin S, Bykov VJ, Wiman KG. Synergistic rescue of nonsense mutant tumor suppressor p53 by combination treatment with aminoglycosides and Mdm2 inhibitors. Front Oncol 2018; 7: 323. <https://doi.org/10.3389/fonc.2017.00323>
8. Floquet C, Hatin I, Rousset J-P, Bidou L. Statistical analysis of readthrough levels for nonsense mutations in mammalian cells reveals a major determinant of response to gentamicin. PLoS Genet 2012; 8: e1002608. doi:10.1371/journal.pgen.1002608
9. Bordeira-Carriço R, Pêgo AP, Santos M, Oliveira C. Cancer syndromes and therapy by stop-codon readthrough. Trends Mol Med 2012; 18: 667-678. [https://doi.org/10.1016/j.molmed.2012.09.004](https://doi.org/10.1016/j.molmed.2012.09.004)
10. Prokhorova I, Altman RB, Djumagulov M, Shrestha JP, Urzhumtsev A, Ferguson A, Chang CWT, Yusupov M, Blanchard SC, Yusupova, G. Aminoglycoside interactions and impacts on the eukaryotic ribosome. Proc Nat Acad Sci 2017; 114: E10899-E10908. <https://doi.org/10.1073/pnas.1715501114>
11. Palmer E, Wilhelm JM, Sherman F. Phenotypic suppression of nonsense mutants in yeast by aminoglycoside antibiotics. Nature 1979; 277: 148–150.28. DOI: [10.1038/277148a0](https://doi.org/10.1038/277148a0)
12. Miftakhova RR, Akhunzyanov A, Filina JV, Khaiboullina SF, Rizvanov AA. Antibiotics target MCF-7 breast cancer stem cells in hypoxic environment. J Clin Oncol 2017; 35:15_suppl, e14068-e14068 DOI: 10.1200/JCO.2017.35.15_suppl.e14068
13. Chlebda E, Merwid-Lad A, Szumny D, Trocha M, Fereniec-Golebiewska L, Gêbarowska E, Kowalski P, Szelag A.

Antitumor effect of macrolides – erythromycin and roxithromycin in B16 melanoma-transplanted mice. Pharmacol Rep 2007; 59 (Suppl 1): 269-274. ISSN 1734-1140

1. Hamada K, Kita E, Sawaki M, Mikasa K, Narita N. Antitumor effect of erythromycin in mice. Chemotherapy 1995; 41: 59-69. <https://doi.org/10.1159/000239325>
2. Pillozzi S, Masselli M, Gasparoli L, D'amico M, Polletta L, Veltroni M, Favre C, Basso G, Becchetti A, Arcangeli A. Macrolide antibiotics exert antileukemic effects by modulating the autophagic flux through inhibition of hERG1 potassium channels. Blood Cancer J 2016; 6: e423. DOI: [10.1038/bcj.2016.32](https://doi.org/10.1038/bcj.2016.32)
3. Ishii A, Kumasaka M, Nagashima Y, Nakajima Y, KuramochiK, Sugawara F, Narukawa M, Kamakura T. A eukaryotic molecular target candidate of roxithromycin: fungal differentiation as a sensitive drug target analysis system. Biosci Biotechnol Biochem 2013; 77: 1539-1547. <https://doi.org/10.1271/bbb.130210>
4. Scholl UI, Abriola L, Zhang C, Reimer EN, Plummer M, Kazmierczak BI, Zhang J, Hoyer D, Merkel JS, Wang W, Lifton RP. Macrolides selectively inhibit mutant KCNJ5 potassium channels that cause aldosterone-producing adenoma. J Clin Invest 2017; 127: 2739-2750. <https://doi.org/10.1172/JCI91733>.
5. Yatsunami, J., Fukuno, Y., Nagata, M., Tominaga, M., Aoki, S., Tsuruta, N., Kawashima M, Taniguchi S, Hayashi, S. I. Antiangiogenic and antitumor effects of 14-membered ring macrolides on mouse B16 melanoma cells. Clin Exp Metastasis 1999; 17: 359-365. DOI: [10.1023/a:1006605725619](https://doi.org/10.1023/a:1006605725619)
6. Van Nuffel AM, Sukhatme V, Pantziarka P, Meheus L, Sukhatme VP, Bouche G. Repurposing drugs in oncology (ReDO)—clarithromycin as an anti-cancer agent. Ecancer 2015; 9: 513 DOI: 10.3332/ecancer.2015.51
7. Ferreri AJ, Sassone M, Kiesewetter B, Govi S, Scarfò L, Donadoni G, Raderer,M. High-dose clarithromycin is an active monotherapy for patients with relapsed/refractory extranodal marginal zone lymphoma of mucosa-associated lymphoid tissue (MALT): the HD-K phase II trial. Ann Oncol 2015; 26: 1760-1765. <https://doi.org/10.1093/annonc/mdv214>
8. Ferreri AJM, Cecchetti C, Kiesewetter B, Sassone M, Calimeri T, Perrone S, Ponzoni M, Raderer M. Clarithromycin as a "repurposing drug" against MALT lymphoma. Brit J Haematol 2018; 182: 913-915. <https://doi.org/10.1111/bjh.14878>
9. Govi S, Dognini GP, Licata G, Crocchiolo R, Resti AG, Ponzoni M, Ferreri AJ. Six‐month oral clarithromycin regimen is safe and active in extranodal marginal zone B‐cell lymphomas: final results of a single‐centre phase II trial. Brit J Haematol 2010; 150: 226–229.
10. Musto P, Falcone A, Sanpaolo G, Bodenizza C, Carotenuto M, Corella AM. Inefficacy of clarithromycin in advanced multiple myeloma: A definitive report [1]. Haematologica 2002; 87: 658-659. PMID: 12031924
11. Moreau P, Huynh A, Facon T, Bouilly I, Sotto JJ, Legros L, Milpied N, Attal M, Bataille R, Harousseau JL, du Myélome IF. Lack of efficacy of clarithromycin in advanced multiple myeloma. Leukemia 1999; 13: 490-491. DOI: [10.1038/sj/leu/2401167](https://doi.org/10.1038/sj/leu/2401167)
12. Mukai S, Moriya S,Hiramoto M, Kazama H, Kokuba H, Che XF, Yokoyama T, Sakamoto S, Sugawara A, Sunazuka T, Ömura S, Handa H, Itoi T, Miyazawa K. Macrolides sensitize EGFR-TKI-induced non-apoptotic cell death via blocking autophagy flux in pancreatic cancer cell lines. Int J Oncol 2016; 48: 45-54. DOI: 10.3892/ijo.2015.3237
13. Moriya S, Che X‑F, Komatsu S, Abe A, Kawaguchi T, Gotoh A, Inazu M, Tomoda A, Miyazawa K. Macrolide antibiotics block autophagy flux and sensitize to bortezomib via endoplasmic reticulum stress‑mediated CHOP induction in myeloma cells. Int J Oncol 2013; 42:1541–50. https ://doi.org/10.3892/ijo.2013.1870.
14. Hirasawa K, Moriya S, Miyahara K, Kazama H, Hirota A, Takemura J, Abe A, Inazu M, Hiramoto M, Tsukahara K, Miyazawa, K. Macrolide antibiotics exhibit cytotoxic effect under amino acid-depleted culture condition by blocking autophagy flux in head and neck squamous cell carcinoma cell lines. PloS one 2016; 11(12), e0164529. DOI: [10.1371/journal.pone.0164529](https://doi.org/10.1371/journal.pone.0164529)
15. Qiao X, Wang X, Shang Y, Li Y, Chen SZ. Azithromycin enhances anticancer activity of TRAIL by inhibiting autophagy and up-regulating the protein levels of DR4/5 in colon cancer cells in vitro and in vivo. Cancer Commun 2018; 38: 43. <https://doi.org/10.1186/s40880-018-0309-9>
16. Zhou X, Zhang Y, Li Y, Hao X, Liu X, Wang Y. Azithromycin synergistically enhances anti-proliferative activity of vincristine in cervical and gastric cancer cells. Cancers 2012; 4: 1318-1332. doi:10.3390/cancers4041318
17. Li F, Huang J, Ji D, Meng Q, Wang C, Chen S, Wang X, Zhu Z, Hang C, Shi Y, Liu S, Liu, C. Azithromycin effectively inhibits tumor angiogenesis by suppressing vascular endothelial growth factor receptor 2‑mediated signaling pathways in lung cancer. Oncol Lett 2017; 14: 89-96. <https://doi.org/10.3892/ol.2017.6103>
18. Tian F, Wang C, Tang M, Li J, Cheng X, Zhang S, .Huang Y, Li H. The antibiotic chloramphenicol may be an effective new agent for inhibiting the growth of multiple myeloma. Oncotarget 2016; 7: 51934. doi: [10.18632/oncotarget.10623](https://dx.doi.org/10.18632%2Foncotarget.10623)
19. Leiter LM, Thatte HS, Okafor C, Marks PW, Golan DE, Bridges KR. Chloramphenicol‐induced mitochondrial dysfunction is associated with decreased transferrin receptor expression and ferritin synthesis in K562 cells and is unrelated to IRE‐IRP interactions. J Cell Physiol 1999; 180: 334-344.

[https://doi.org/10.1002/(SICI)1097-4652(199909)180:3<334::AID-JCP4>3.0.CO;2-Q](https://doi.org/10.1002/(SICI)1097-4652(199909)180:3%3c334::AID-JCP4%3e3.0.CO;2-Q)

1. Li CH, Cheng YW, Liao PL, Yang YT, Kang JJ. Chloramphenicol causes mitochondrial stress, decreases ATP biosynthesis, induces matrix metalloproteinase-13 expression, and solid-tumor cell invasion. Toxicol Sci 2010; 116: 140-150. <https://doi.org/10.1093/toxsci/kfq085>
2. Li CH, Tzeng SL, Cheng YW, Kang JJ. Chloramphenicol-induced mitochondrial stress increases p21 expression and prevents cell apoptosis through a p21-dependent pathway. J Biol Chem 2005; 280: 26193-26199. DOI: 10.1074/jbc.M501371200
3. Zhu Y, Weldon JE. Evaluating the influence of common antibiotics on the efficacy of a recombinant immunotoxin in tissue culture. BMC Res Notes 2019; 12: 293. https://doi.org/10.1186/s13104-019-4337-6
4. Esner M, Graifer D, Lleonart ME, Lyakhovich A. Targeting cancer cells through antibiotics-induced mitochondrial dysfunction requires autophagy inhibition. Cancer Lett 2017; 384: 60-69. [https://doi.org/10.1016/j.canlet.2016.09.023](https://doi.org/10.1016/j.canlet.2016.09.023)
5. Abad E, García-Mayea Y, Mir C, Sebastian D, Zorzano A, Potesil D, Zdrahal Z, Lyakhovich A, Lleonart, M E. Common metabolic pathways implicated in resistance to chemotherapy point to a key mitochondrial role in breast cancer. Mol Cell Proteom 2019; 18: 231-244. <https://doi.org/10.1074/mcp.RA118.001102>
6. Sharon D, Cathelin S, Mirali S, Di Trani JM, Yanofsky DJ, Keon KA, Rubinstein JL, Schimmer AD, Ketela T, Chan SM. 2019. Inhibition of mitochondrial translation overcomes venetoclax resistance in AML through activation of the integrated stress response. Sci Translat Med 2019; 11(516) pii: eaax2863. DOI: 10.1126/scitranslmed.aax2863
7. Kroon AM, Dontje BH, Holtrop M, Van Den Bogert C. The mitochondrial genetic system as a target for chemotherapy: tetracyclines as cytostatics. Cancer Lett 1984; 25: 33-40. [https://doi.org/10.1016/S0304-3835(84)80023-3](https://doi.org/10.1016/S0304-3835(84)80023-3)
8. Aaron D Schimmer AD, Škrtić M. Therapeutic potential of mitochondrial translation inhibition for treatment of acute myeloid leukemia, Expert Rev Hematol 2012; 5: 117-119. <https://doi.org/10.1586/ehm.12.8>
9. Chatzispyrou IA, Held NM, Mouchiroud L, Auwerx J, Houtkooper RH. Tetracycline antibiotics impair mitochondrial function and its experimental use confounds research. Cancer Res 2015; 75: 4446-4449. DOI: [10.1158/0008-5472.CAN-15-1626](https://doi.org/10.1158/0008-5472.CAN-15-1626)
10. Moullan N, Mouchiroud L, Wang X, Ryu D, Williams EG, Mottis A, Jovaisaite V, Frochaux MV, Quiros PM, Deplacke B, Houtkooper RH, Auwerx J. Tetracyclines disturb mitochondrial function across eukaryotic models: a call for caution in biomedical research. Cell Reports 2015; 10: 1681-1691. [https://doi.org/10.1016/j.celrep.2015.02.034](https://doi.org/10.1016/j.celrep.2015.02.034)
11. Wang B, Ao J, Yu, D, Rao T, Ruan Y, Yao X. Inhibition of mitochondrial translation effectively sensitizes renal cell carcinoma to chemotherapy. Biochem Biophys Res Commun 2017; 490: 767-773. [https://doi.org/10.1016/j.bbrc.2017.06.115](https://doi.org/10.1016/j.bbrc.2017.06.115)
12. Koltai T. Tetracyclines against cancer. A review. Unpublished 2015. Available at: <https://doi.org/10.13140/rg.2.1.4097.1600>
13. Norberg E, Lako A, Chen PH, Stanley IA, Zhou F, Ficarro SB., Chapuy B, Chen L, Rodig S, Shin D, Choi DW, Lee S, Shipp MA, Marto JA, Danial NN. Differential contribution of the mitochondrial translation pathway to the survival of diffuse large B-cell lymphoma subsets. Cell Death Differ 2017; 24: 251-262. DOI: 10.1038/cdd.2016.116
14. MatsumotoT, Uchiumi T, Monji K, Yagi M, Setoyama D, Amamoto R, Matsushima Y, Shiota M, Eto M, Kang D. Doxycycline induces apoptosis via ER stress selectively to cells with a cancer stem cell-like properties: importance of stem cell plasticity. Oncogenesis 2017; 6: 1-11. DOI: 10.1038/s41389-017-0009-3
15. Zhang L, Xu L, Zhang F, Vlashi E. Doxycycline inhibits the cancer stem cell phenotype and epithelial-to-mesenchymal transition in breast cancer. Cell Cycle 2017; 16: 737-745. <https://doi.org/10.1080/15384101.2016.1241929>
16. Lin CC, Lo MC, Moody RR, Stevers NO, Tinsley SL, Sun D. Doxycycline targets aldehyde dehydrogenase‑positive breast cancer stem cells. Oncol Rep 2010; 39: 3041-3047. <https://doi.org/10.1080/15384101.2016.1241929>
17. Zhu C, Yan X, Yu A, Wang Y. Doxycycline synergizes with doxorubicin to inhibit the proliferation of castration-resistant prostate cancer cells. Acta Bioch Bioph Sin 2017; 49: 999-1007. DOI: [10.1093/abbs/gmx097](https://doi.org/10.1093/abbs/gmx097)
18. Fife RS, Sledge Jr GW. Effects of doxycycline on cancer cells in vitro and in vivo. Adv Dent Res 1998; 12: 94-96. DOI: [10.1177/08959374980120012801](https://doi.org/10.1177/08959374980120012801)
19. Scatena C, Roncella M, Di Paolo A, Aretini P, Menicagli M, Fanelli G, Marini C, Mazzanti CM, Ghill M, Sotiga F, Lisanti MP, Naccarato AG. Doxycycline, an inhibitor of mitochondrial biogenesis, effectively reduces cancer stem cells (CSCs) in early breast cancer patients: a clinical pilot study. Front Oncol 2018; 8: 452. <https://doi.org/10.3389/fonc.2018.00452>
20. De Francesco EM, Maggiolini M, Tanowitz HB, Sotgia F, Lisanti MP. Targeting hypoxic cancer stem cells (CSCs) with doxycycline: implications for optimizing anti-angiogenic therapy. Oncotarget 2017; 8: 56126. DOI: [10.18632/oncotarget.18445](https://dx.doi.org/10.18632%2Foncotarget.18445)
21. Song H, Fares M, Maguire KR, Siden A, Potacova Z. Cytotoxic effects of tetracycline analogues (doxycycline, minocycline and COL-3) in acute myeloid leukemia HL-60 cells. PLoSONE 2014; 9: e114457. DOI:10.1371/journal.pone.0114457
22. Lamb R, Fiorillo M, Chadwick A, Ozsvari B, Reeves KJ, Smith DL, Clarke RB, Howell SJ, Cappello AR, Martinez-Outschoorn UE, Peiris-Pages M, Sotgia F, Lisanti MP. Doxycycline down-regulates DNA-PK and radiosensitizes tumor initiating cells: Implications for more effective radiation therapy. Oncotarget. 2015; 6:14005–14025. DOI: [10.18632/oncotarget.4159](https://dx.doi.org/10.18632%2Foncotarget.4159)
23. Peiris-Pagès M, Sotgia F, Lisanti MP. Doxycycline and therapeutic targeting of the DNA damage response in cancer cells: old drug, new purpose. Oncoscience 2015; 2: 696-699. doi: [10.18632/oncoscience.215](https://dx.doi.org/10.18632%2Foncoscience.215)
24. Yoon JM, Koppula S, Huh SJ, Hur SJ, Kim CG. Low concentrations of doxycycline attenuates FasL-induced apoptosis in HeLa cells. Biol Res 2015; 48: 38. DOI 10.1186/s40659-015-0025-8
25. Wang SQ, Zhao BX, Liu Y, Wang YT, Liang QY, Cai Y, Zhang YQ, Yang JH, Song ZH, Li GF. New application of an old drug: Antitumor activity and mechanisms of doxycycline in small cell lung cancer. Int J Oncology 2016; 48: 1353-1360.    <https://doi.org/10.3892/ijo.2016.3375>
26. Xu Z, Yan Y, Li Z, Qian L, Gong Z. The antibiotic drug tigecycline: a focus on its promising anticancer properties. Front Pharmacol 2016; 7:473. doi: [10.3389/fphar.2016.00473](https://dx.doi.org/10.3389%2Ffphar.2016.00473)
27. Dong Z, Abbas MN, Kausar S, Yang , Li, L, Tan L, Cui H. 2019. Biological functions and molecular mechanisms of antibiotic tigecycline in the treatment of cancers. Int J Mol Sci 2019; 20: 3577. <https://doi.org/10.3390/ijms20143577>
28. Arora R, Jain S, Rahimi H. Evaluating the efficacy of tigecycline to target multiple cancer-types: A Review. STEM Fellowship Journal 2019; 4: 5-11. <https://doi.org/10.17975/sfj-2018-002>
29. Yadav V, Talwar P. Repositioning of fluoroquinolones from antibiotic to anti-cancer agents: An underestimated truth. Biomed Pharmacother 2019; 111: 934-946. [https://doi.org/10.1016/j.biopha.2018.12.119](https://doi.org/10.1016/j.biopha.2018.12.119)
30. Gurtovska N, Kloskowski T, Drewa T. Ciprofloxacin criteria in antimicrobial prophylaxis and bladder cancer recurrence. Med Sci Monit 2010; 16: RA218-223. PMID: 20885364
31. Idowu T, Schweizer F. Ubiquitous nature of fluoroquinolones: the oscillation between antibacterial and anticancer activities. Antibiotics 2017; 6: 26. <https://doi.org/10.3390/antibiotics6040026>
32. Alibek K, Bekmurzayeva A, Mussabekova A, Sultankulov B. Using antimicrobial adjuvant therapy in cancer treatment: a review. Infect Agents Cancer 2012; 7: 33. DOI: [10.1186/1750-9378-7-33](https://doi.org/10.1186/1750-9378-7-33)
33. Zehavi-Willner T, Shalit I. The inhibitory effect of ciprofloxacin on proliferation of a murine bladder carcinoma cell line. J Antimicrob Chemother 1992; 29: 323-328. <https://doi.org/10.1093/jac/29.3.323>
34. Shalit I, Nasrallah N, Bar-On S, Rabau M. In vitro effect of ciprofloxacin and ofloxacin on murine and human colon carcinoma cell lines. Drugs 1995; 49: 296-297. DOI: [10.2165/00003495-199500492-00076](https://doi.org/10.2165/00003495-199500492-00076)
35. Hahn T, Barak Y, Liebovich E, Malach L, Dagan O, Rubinstein E. Ciprofloxacin inhibits human hematopoietic cell growth: synergism with tumor necrosis factor and interferon. Exp Hematol 1991; 19: 157–160. PMID: 1899831
36. Jaber DF, Jallad MAN, Abdelnoor AM. The effect of ciprofloxacin on the growth of B16F10 melanoma cells. J Cancer Res Ther 2017; 13: 956-960. DOI**:** 10.4103/0973-1482.180610
37. Zandi A, Moini Zanjani T, Ziai SA, Khazaei Poul Y, Haji Molla Hoseini M. Evaluation of the Cytotoxic Effects of Ciprofloxacin on Human Glioblastoma A-172 Cell Line. Middle East J Cancer 2017; 8: 119-126. Available at: http://mejc.sums.ac.ir/article_42078_a46cd593ec34ec5c87ad24f1a74bbbcb.pdf
38. Esmaeilzadeh A, Ebtekar M, Biglari A, Hassan ZM. Influence of ciprofloxacin on glioma cell line GL26: A new application for an old antibiotic. African J Microbiol Res 2012; 6: 4891-4896. Available at: <http://www.academicjournals.org/AJMR>
39. Miclau T, Edin ML, Lester GE, Lindsey RW, Dahners LE. 1998. Effect of ciprofloxacin on the proliferation of osteoblast‐like MG‐63 human osteosarcoma cells in vitro. J Orthop Res 1998; 16: 509-512. <https://doi.org/10.1002/jor.1100160417>
40. Kloskowski T, Olkowska J, Nazlica A, Drewa T. The influence of ciprofloxacin on hamster ovarian cancer cell line CHO AA8. Acta Pol Pharm 2010; 67: 345-349. PMID: 20635529
41. Lim EJ, Yoon YJ, Heo J, Lee TH, Kim YH. Ciprofloxacin enhances TRAIL-induced apoptosis in lung cancer cells by upregulating the expression and protein stability of death receptors through CHOP expression. Int J Mol Sci 2018; 19: 3187. <https://doi.org/10.3390/ijms19103187>
42. Koziel R, Szczepanowska J, Magalska A, Piwocka K, Duszynski J, Zablocki K. Ciprofloxacin inhibits proliferation and promotes generation of aneuploidy in Jurkat cells. J Physiol Pharmacol 2010; 61: 233-239. PMID: 20436225
43. Gafter-Gvili A, Fraser A, Paul M, Leibovici L. Meta-analysis: antibiotic prophylaxis reduces mortality in neutropenic patients. Ann Intern Med 2005; 142:979–995. DOI: 10.7326/0003-4819-142-12_Part_1-200506210-00008
44. Paul M, Gafter-Gvili A, Fraser A, Leibovici L. The anti-cancer effects of quinolone antibiotics. Eur J Clin Microbiol Infect Dis 2007; 26: 825–831. DOI: 10.1007/s10096-007-0375-4
45. Koltai T. Is ciprofloxacin an anti-cancer drug? A minireview. 2016. Available at: <https://www.researchgate.net/publication/305319162>. DOI: [10.13140/RG.2.1.3255.1920](https://www.researchgate.net/deref/http%3A%2F%2Fdx.doi.org%2F10.13140%2FRG.2.1.3255.1920?_sg%5B0%5D=k2bWSNQrzXCPX-obEigErSDYC-tMmvNhb2WOTWMVscM3lFqFcrzsm6lhSMcXyPxqoAGR_IMh408Pz4yy-QYK4B-r1Q.KVGisslgn60JtBdyz8-PPKIyJWcMDa4T1YOKvkxz7gL7VRvqY9gCbOoYk5G-d54-Pq-BNaorgZhUU1b4AJqF5A)
46. Yadav V, Varshney P, Sultana S, Yadav J, Saini N. Moxifloxacin and ciprofloxacin induces S-phase arrest and augments apoptotic effects of cisplatin in human pancreatic cancer cells via ERK activation. BMC Cancer 2015; 15: 581. DOI 10.1186/s12885-015-1560-y
47. Yadav V, Sultana S, Yadav J, Saini N. Gatifloxacin induces S and G2-phase cell cycle arrest in pancreatic cancer cells via p21/p27/p53. PLoS One 2012; 7(10). DOI: [10.1371/journal.pone.0047796](https://dx.doi.org/10.1371%2Fjournal.pone.0047796)
48. Song H, Wu H, Wu S, Ge T, Wang G, Zhou Y, Sheng S, Jiang J. Antibiotic levofloxacin inhibitds proliferation and induces apoptosis of lung cancer cells through inducing mitochondrial dysfunction and oxidative stress. Biomed Pharmacother 2016; 84: 1137-1143. DOI : 10.1016/j.biopha.2016.10.034
49. Zhang Q, Zhang C, Xi Z. Enhancement of RNAi by a small molecule antibiotic enoxacin. Cell Res 2008; 18: 1077-1079. DOI: 10.1038/cr.2008.287
50. Li Y, Ji P, Jin P. Probing the microRNA pathway with small molecules. Bioorgan Med Chem 2013; 21: 6119-6123. [https://doi.org/10.1016/j.bmc.2013.05.030](https://doi.org/10.1016/j.bmc.2013.05.030)
51. Shan G, Li Y, Zhang J, Li W, Szulwach KE, Duan R, Faghihi MA, Khalil AM, Lu L, Paroo Z, Chan AWS, Shi Z, Liu Q, Wahlestedt C, He C, Jin P. A small molecule enhances RNA interference and promotes microRNA processing. Nat Biotechnol 2008; 26: 933-940. DOI:10.1038/nbt.1481
52. Toro EJ, Zuo J, Ostrov DA, Catalfamo D, Bradaschia-Correa V, Arana-Chavez V, Caridad AR, Neubert JK, Wronski TJ, Wallert SM, Holliday LS. Enoxacin directly inhibits osteoclastogenesis without inducing apoptosis. J Biological Chem 2012; 287: 17894-17904. DOI 10.1074/jbc.M111.280511
53. Toro EJ, Zuo J, Guiterrez A, La Rosa RL, Gawron AJ, Bradaschia-Correa V, Arana-Chavez V, Dolce C, Rivera MF, Kesavalu L, Bhattacharyya I, Neubert JK, Holliday LS. Bis-enoxacin inhibits bone resorption and orthodontic tooth movement. J Dent Res 2013; 92: 925-931. [https://doi.org/10.1177/0022034513501876](https://doi.org/10.1177%2F0022034513501876)
54. Toro E, Ostrov DA, Wronski TJ, Holliday LS. Rational Identification of Enoxacin as a Novel V-ATPase-Directed Osteoclast Inhibitor. Current Protein and Peptide Science 2012; 13:180-191. DOI: [10.2174/138920312800493151](https://www.researchgate.net/deref/http%3A%2F%2Fdx.doi.org%2F10.2174%2F138920312800493151?_sg%5B0%5D=j25iecSGC5W_0GV2RfhVXwFl6v86t92PbzjEBcMRZNGvdp8e7usQfpzHpVaaLxT7GGJO4m73jWD3Azq1zIugn_kj2g.lYwNTcRJldqOzdAt1pxs2VOEj0TNsXApzJ8XZMFAIgDQZEDj9s5n1jQZeuhQAf6RtU_robi98L2rxheTfAveJQ)
55. Ostrov DA, Magis AT, Wronski TJ, Chan EK, Toro EJ, Donatelli RE, Sajek K, Haroun IN, Nagib MI, Piedrahita A, Harris A, Holliday LS. Identification of enoxacin as an inhibitor of osteoclast formation and bone resorption by structure-based virtual screening. J Med Chem 2009; 52: 5144–5151. DOI: [10.1021/jm900277z](https://doi.org/10.1021/jm900277z)
56. Gioia U, Francia S, Cabrini M, Brambillasca S, Michelini F, Jones-Weinert CW, di Fagagna FDA. Pharmacological boost of DNA damage response and repair by enhanced biogenesis of DNA damage response RNAs. Sci Rep 2019; 9: 6460. DOI: [10.1038/s41598-019-42892-6](https://doi.org/10.1038/s41598-019-42892-6)
57. Melo S, Villanueva A, Moutinho C, Davalos V, Spizzo R, Ivan C, Rossi S, Setien F, Casanovas O, Simo-Riudalbas L, Carmona J, Carrere J, Vidal A, Aytes A, Puertas S, Ropero S, Kalluri R, Croce CM, Calin GA, Esteller M. Small molecule enoxacin is a cancer-specific growth inhibitor that acts by enhancing TAR RNA-binding protein 2-mediated microRNA processing. Proc Nat Acad Sci 2011; 108: 4394-4399. <https://doi.org/10.1073/pnas.1014720108>
58. Sousa EJ, Graça I, Baptista T, Vieira FQ, Palmeira C, Henrique R, Jerónimo C. Enoxacin inhibits growth of prostate cancer cells and effectively restores microRNA processing. Epigenetics 2013; 8: 548-558. DOI: 10.3390/molecules24081580
59. Valianatos G, Valcikova B, Growkova K, Verlande A, Mlcochova J, Radova L, Stetkova M, Vyhnakova M, Slaby O, Uldrijan S. A small molecule drug promoting miRNA processing induces alternative splicing of MdmX transcript and rescues p53 activity in human cancer cells overexpressing MdmX protein. PloS one 2017; 12(10), e0185801.  <https://doi.org/10.1371/journal.pone.0185801>
60. Sadowska A, Prokopiuk S, Miltyk W, Surażyński A, Konończuk J, Sawicka D, Car H. Metronidazole affects breast cancer cell lines. Adv Med Sci 2013; 58: 90-95. DOI: 10.2478/v10039- 012- 0070 -2
61. Shim JS, Matsui Y, Bhat S, Nacev BA, Xu J, Bhang HEC, Dhara S, Han KC, Chong CR, Pomper MG, So A, Liu JO. Effect of nitroxoline on angiogenesis and growth of human bladder cancer. J Nat Cancer Inst 2010; 102: 1855-1873. <https://doi.org/10.1093/jnci/djq457>
62. Zhang QI, Wang S, Yang D, Pan K, LiL, Yuan S. Preclinical pharmacodynamic evaluation of antibiotic nitroxoline for anticancer drug repurposing. Oncol Lett 2016; 11: 3265-3272. https://doi.org/10.3892/ol.2016.4380
63. Chan-on W, Huyen NTB, Songtawee N, Suwanjang W, Prachayasittikul S, Prachayasittikul V. Quinoline-based clioquinol and nitroxoline exhibit anticancer activity inducing FoxM1 inhibition in cholangiocarcinoma cells. Drug Des Devel Ther 2015; 9: 2033-2047. DOI: [10.2147/DDDT.S79313](https://dx.doi.org/10.2147%2FDDDT.S79313)
64. Lazovic J, Guo L, Nakashima J, Mirsadraei L, Yong W, Kim HJ, Ellingson B, Wu H, Pope WB. Nitroxoline induces apoptosis and slows glioma growth in vivo. Neuro-Oncology 2015; 17: 53-62. <https://doi.org/10.1093/neuonc/nou139>
65. Yu JG, Ji CH, Shi MH. Nitroxoline induces cell apoptosis by inducing MDM2 degradation in small‐cell lung cancer. Kaohsiung J Med Sci 2019; 35: 202-208. DOI: 10.1002/kjm2.12051
66. Fiorillo M, Lamb R, Tanowitz HB, Cappello AR, Martinez-Outschoorn UE, Sotgia F, Lisanti MP. Bedaquiline, an FDA-approved antibiotic, inhibits mitochondrial function and potently blocks the proliferative expansion of stem-like cancer cells (CSCs). Aging 2016; 8: 1593-1607. [https://doi.org/10.18632/aging.100983](https://doi.org/10.18632/aging.100983)
67. Wu X, Li F, Wang X, Li C, Meng Q, Wang C, Huang J, Chen S, Zhu Z. Antibiotic bedaquiline effectively targets growth, survival and tumor angiogenesis of lung cancer through suppressing energy metabolism. Biochem Biophys Res Commun 2018; 495: 267-272. [https://doi.org/10.1016/j.bbrc.2017.10.136](https://doi.org/10.1016/j.bbrc.2017.10.136)
68. Santavirta S, Takagi M, Kontinen YT, Sorsa T, Suda A. Inhibitory effect of cephalothin on matrix metalloproteinase activity around loose hip prosthesis. Antimicrob Agents Chemother 1996; 40: 244-246. DOI: [10.1128/aac.40.1.244](https://doi.org/10.1128/aac.40.1.244)
69. Georgopapadakou NH. Does cephalothin inhibit matrix metalloproteinases? Antimicrol Agents Chemother 1996; 40: 1969-1970. DOI: [10.1128/AAC.40.8.1969](https://www.researchgate.net/deref/http%3A%2F%2Fdx.doi.org%2F10.1128%2FAAC.40.8.1969?_sg%5B0%5D=p-3kMLqgdSbfmAb5_7T1uXjffe4nKy5wTj8gp3sEA8Tqb7aceKuqD6T1Hp2pMbXU_CUDFT65Uh_N6Hq_cQsCRfpuXQ.Uf4PqH9PcuP3OPSnjg_dXnZiRPl4aF7Gd_25DqTJNvS5yb-qo-HmvTcsiGpRLmjsA7IB83kJkFDACJPdfn1lNQ)
70. Cifcibasi E, Kantarci A, Badur S, Issever H, Cintan S. Impact of metronidazole and amoxicillin combination on matrix metalloproteinases-1 and tissue inhibitors of matrix metalloproteinases balance in generalized aggressive periodontitis. Eur J Dent 2015; 9: 53-59. DOI: 10.4103/1305-7456.149642
71. Kobayashi Y, Wada H, Rossios C, Takagi D, Higaki M, Mikura S, Goto H, Barnes PJ,Ito K. A novel macrolide solithromycin exerts superioranti-inflammatory effect via NF-kB inhibition. J Pharmacol Exp Ther 2013; 345: 76-84. DOI: <https://doi.org/10.1124/jpet.112.200733>
72. Asano K, Hisamitsu T, Suzaki H. Suppression of matrix metalloproteinase production from nasal fibroblasts by macrolide antibiotics in vitro. Eur Respir J 2004; 23: 671-678. DOI: [10.1183/09031936.04.00057104](https://doi.org/10.1183/09031936.04.00057104)
73. Kanai KI, Asano K, Hisamitsu T, Suzaki H. Suppression of matrix metalloproteinase-9 production from neutrophils by a macrolide antibiotic, roxithromycin, in vitro. Mediat Inflamm 2004; 13: 313-319. [https://doi.org/10.1080/09629350400008810](https://doi.org/10.1080/09629350400008810)
74. Ogawa M, Suzuki JI, Hishikari K, Takayama K, Tanaka H, Isobe M. Clarithromycin attenuates acute and chronic rejection via matrix metalloproteinase suppression in murine cardiac transplantation. J Am Coll Cardiol 2008; 51: 1977-1985. DOI: 10.1016/j.jacc.2008.01.050
75. Ogawa M, Suzuki J, Takayama K, Isobe M. Matrix metalloproteinase suppression induced by clarithromycin in murine cardiac allografts. Transplantat P 2009; 41: 395-397. [https://doi.org/10.1016/j.transproceed.2008.10.057](https://doi.org/10.1016/j.transproceed.2008.10.057)
76. Suzuki JI, Ogawa M, Hishikari K, Watanabe R, Takayama K, Hirata Y, Nagai R, Isobe M. Novel effects of macrolide antibiotics on cardiovascular diseases. Cardiovas Ther 2012; 30: 301-307. DOI: 10.1111/j.1755-5922.2011.00303.x
77. Simpson JL, Powell H, Boyle MJ, Scott RJ, Gibson PG. Clarithromycin targets neutrophilic airway inflammation in refractory asthma. Am JRespir Crit Care Med 2008; 177: 148-155. DOI: [10.1164/rccm.200707-1134oc](https://doi.org/10.1164/rccm.200707-1134oc)
78. Ribeiro CMP, Hurd H, Wu Y, Martino MEB, Jones L, Brighton B, Boucher RC, O’Neal WK. Azithromycin treatment alters gene expression in inflammatory, lipid metabolism, and cell cycle pathways in well-differentiated human airway epithelia. PLoS ONE 2009; 4(6): e5806. DOI:10.1371/journal.pone.0005806
79. Singh S, Kubler A, Singh UK, Singh A, Gardiner H, Prasad R, Elkington PT, Friedland JS. Antimycobacterial drugs modulate immunopathogenic matrix metalloproteinases in a cellular model of pulmonary tuberculosis. Antimicrob Agents Chemother 2014; 58: 4657-4665. DOI: [10.1128/AAC.02141-13](https://dx.doi.org/10.1128%2FAAC.02141-13)
80. Murphy DM, Forrest IA, Ward C, Corris PA, Johnson GE, Jones D, Fisher AJ. Effect of azithromycin on primary bronchial epithelial cells derived from stable lung allografts. Thorax 2007; 62: 834-835. [http://dx.doi.org/10.1136/thx.2007.077818](http://dx.doi.org/10.1136/thx.2007.077818)
81. Vanaudenaerde BM, Wuyts WA, Dupont LJ, Van Raemdonck DE, Demedts MM,Verleden GM. Interleukin-17 stimulates release of interleukin-8 by human airway smooth muscle cells in vitro: a potential role for interleukin-17 and airway smoothmuscle cells in bronchiolitis obliterans syndrome. J Heart Lung Transplant 2003; 22:1280–1283. [https://doi.org/10.1016/S1053-2498(02)01234-2](https://doi.org/10.1016/S1053-2498(02)01234-2)
82. Vandooren J, KnoopsS, Aldinucci-Buzzo JL, Boon L, Martens E, Opdenakker G, Kolaczkowska E. 2017. Differentialinhibitionof activity,activationand geneexpressionof MMP-9 in THP-1cells byazithromycinand minocyclineversusbortezomib:A comparativestudy.PLOSONE 2017; 12(4):e0174853. https://doi.org/10.1371/journal.pone.0174853
83. Fernandez-Robredo P, Recalde S, Moreno-Orduña M, García-García L, Zarranz-Ventura J, García-Layana A. Azithromycin reduces inflammation in a rat model of acute conjunctivitis. Mol Vis 2013; 19: 153-165. PMID: 23378729
84. Verleden SE, Vandooren J, Vox R, Willems S, Dupont LJ, Verleden GM, van Raemdonck DE, Opdenakker G, Vanaudenaerde BM. Azithromycin decreases MMP-9 expression in the airways of lung transplant recipients. Transpl Immunol 2011; 25: 159-162. [https://doi.org/10.1016/j.trim.2011.06.006](https://doi.org/10.1016/j.trim.2011.06.006)
85. Akinnusi ME, Hattemer A, Gao W, El-Solh AA. Does linezolid modulate lung innate immunity in a murine model of methicillin-resistant Staphylococcus aureus pneumonia?. Crit Care Med 2011; 39: 1944-1952. DOI: 10.1097/CCM.0b013e31821bd79e
86. Kalil AC.Linezolid does not show advantages over vancomycin in modulating the pulmonary immune response: how should we conciliate these new findings with the Zephyr trial results? Crit Care Med 2011; 39:2009-2010. DOI: 10.1097/CCM.0b013e318221741c
87. Hanemaaijer R, van Lent N, Sorsa T, Salo T, Yrgö, Kontinen T, Lindemann J. Inhibition of matrix metalloproteinases (MMPs) by tetracyclines. In: Nelson M, Hillen W, Greenwald RA (eds) Tetracyclines in biology, chemistry and medicine. Birkhäuser, Basel. 2001, pp 267-281. <https://doi.org/10.1007/978-3-0348-8306-1_11>. Print ISBN 978-3-0348-9511-8
88. Liu J, Khalil RA. Matrix metalloproteinase inhibitors as investigational and therapeutic tools in unrestrained tissue remodeling and pathological disorders. Prog Mol Biol Transl Sci 2017; 148: 355-420. [https://doi.org/10.1016/bs.pmbts.2017.04.003](https://doi.org/10.1016/bs.pmbts.2017.04.003)
89. Hidalgo M, Eckhardt SG. Development of matrix metalloproteinase inhibitors in cancer therapy. J Nat Cancer Inst 2001; 93: 178-193. <https://doi.org/10.1093/jnci/93.3.178>
90. Smith GN Jr, Mickler EA, Hasty KA, Brandt KD. 1999. Specificity of inhibition of matrix metalloproteinase activity by doxycycline: relationship to structure of the enzyme. Arthritis Rheum 1999; 42: 1140–1146

[https://doi.org/10.1002/1529-0131(199906)42:6<1140::AID-ANR10>3.0.CO;2-7](https://doi.org/10.1002/1529-0131(199906)42:6%3c1140::AID-ANR10%3e3.0.CO;2-7)

1. Lokeshwar BL. MMP inhibition in prostate cancer. Ann N Y Acad Sci 1999; 878: 271–89. DOI: [10.1111/j.1749-6632.1999.tb07690.x](https://doi.org/10.1111/j.1749-6632.1999.tb07690.x)
2. Fife RS, Rougraff BT, Proctor C, Sledge GW Jr. Inhibition of proliferation and induction of apoptosis by doxycycline in cultured human osteo-sarcoma cells. J Lab Clin Med 1997; 130: 530–534. DOI: [10.1016/s0022-2143(97)90130-x](https://doi.org/10.1016/s0022-2143(97)90130-x)
3. Fife RS, Sledge GW Jr. Effects of doxycycline on in vitro growth, migration, and gelatinase activity of breast carcinoma cells. J Lab Clin Med 1995; 125: 407–411. PMID: 7897308
4. Fife RS, Sledge GW Jr, Roth BJ, Proctor C. Effects of doxycycline on human prostate cancer cells in vitro. Cancer Lett 1998; 127: 37–41. DOI: [10.1016/s0304-3835(98)00003-2](https://doi.org/10.1016/s0304-3835(98)00003-2)
5. Duivenvoorden WC, Hirte H, Singh G. Use of tetracycline as an inhibitor of matrix metalloproteinase activity secreted by human bone-metastasizing cancer cells. Invas Metast 1997; 17: 312–322. PMID: 9949290
6. Sledge GW Jr, Qulali M, Bone EA, Fife R. Effect of matrix metalloproteinase inhibitor batimastat on breast cancer regrowth and metastasis in athymic mice. J Natl Caner Inst 1995; 87: 1546–1550. DOI: [10.1093/jnci/87.20.1546](https://doi.org/10.1093/jnci/87.20.1546)
7. Shlopov BV, Smith Jr GN, Cole AA, Hasty KA. Differential patterns of response to doxycycline and transforming growth factor β1 in the downregulation of collagenases in osteoarthritic and normal human chondrocytes. Arthr Rheum 1999; 42: 719-727. [https://doi.org/10.1002/1529-0131(199904)42:4<719::AID-ANR15>3.0.CO;2-T](https://doi.org/10.1002/1529-0131(199904)42:4%3c719::AID-ANR15%3e3.0.CO;2-T)
8. Modheji M, Olapour S, Khodayar MJ, Jalili A, Yaghooti H. Minocycline is more potent than tetracycline and doxycycline in inhibiting MMP-9 in vitro. Jundishapur J Nat Pharm Prod 2016; 11:e27377. DOI: [10.17795/jjnpp-27377](https://www.researchgate.net/deref/http%3A%2F%2Fdx.doi.org%2F10.17795%2Fjjnpp-27377?_sg%5B0%5D=cjSuuE1VF16XvLO1VK_fzqXAQWB1N-3-FcA4VW6niDQ56zCYEtAsTY5h-nsudxZhZ3Wd2_KxAmfG734Ycry2xsp8Yw.teE9kkTDX5PTdzO2AkiT1Z9_75dQTslRzEtha0WzaTGVp2mqRS7E3I2fyqWHOiImoGtDuBWqBIoNLYTNpbRndQ)
9. Gu Y, Walker C, Ryan ME, Payne JB, Golub LM. Non-antibacterial tetracycline formulations: clinical applications in dentistry and medicine. J Oral Microbiol 2012; 4: 19227. <https://doi.org/10.3402/jom.v4i0.19227>
10. Walker SG, Golub LM. Host modulation therapy for periodontal disease: Subantimicrobial-dose doxycycline, medical as well as dental benefits. Oral Sci 2012; 11: 8. Available at: https://www.oralhealthgroup.com/features/host-modulation-therapy-for-periodontal-disease-subantimicrobial-dose-doxycycline-medical-as-well-as/
11. Golub LM, Lee HM. Periodontal therapeutics: Current host‐modulation agents and future directions. Periodontology 2000. 2020; 82: 186-204. <https://doi.org/10.1111/prd.12315>
12. Golub LM, Wolff M, Roberts S, Lee HM, Leung M, Payonk GS. Treating periodontal diseases by blocking tissue destructive enzymes. J Am Dent Assoc 1994; 125: 163‐169. DOI: [10.14219/jada.archive.1994.0261](https://doi.org/10.14219/jada.archive.1994.0261)
13. Golub LM, Lee HM, Ryan ME, Giannobile WV, Payne JB, Sorsa T. Tetracyclines inhibit connective tissue breakdown by multiple non‐antimicrobial mechanisms. Adv Dent Res 1998; 12:12‐26. [https://doi.org/10.1177/08959374980120010501](https://doi.org/10.1177%2F08959374980120010501)
14. Valentín S, Morales A, Sánchez JL, Rivera A. 2009. Safety and efficacy of doxycycline in the treatment of rosacea. Clin Cosmet Investig Dermatol 2009; 2: 129-140. DOI: [10.2147/ccid.s4296](https://dx.doi.org/10.2147%2Fccid.s4296)
15. Schaller M, Schöfer H, Homey B, Gieler U, Lehmann P, Luger TA, Ruzicka T, Steinhoff M. State of the art: systemic rosacea management. J Dtsch Dermatol Ges 2016; 14: 29-37. DOI: 10.1111/ddg.13141
16. Wladis EJ, Bradley EA, Bilyk JR, Yen MT, Mawn LA. 2016. Oral antibiotics for meibomian gland-related ocular surface disease: a report by the American Academy of Ophthalmology. Ophthalmology 2016; 123: 492-496. http://dx.doi.org/10.1016/j.ophtha.2015.10.062
17. Federici TJ. The non-antibiotic properties of tetracyclines: clinical potential in ophthalmic disease. Pharmacol Res 2011; 64: 614-623. [https://doi.org/10.1016/j.phrs.2011.06.013](https://doi.org/10.1016/j.phrs.2011.06.013)
18. Li DQ, Shang TY, Kim HS, Solomon A, Lokeshwar BL, Pflugfelder SC. Regulated expression of collagenases MMP-1,-8, and-13 and stromelysins MMP-3,-10, and-11 by human corneal epithelial cells. Invest Ophthalmol Vis Sci 2003; 44: 2928-2936. <https://doi.org/10.1167/iovs.02-0874>
19. Smith VA, Cook SD. Doxycycline—a role in ocular surface repair. Brit J Ophthalmol 2004; 88: 619-625. DOI: 10.1136/bjo.2003.025551
20. Castro MM, Kandasamy AD, Youssef N, Schulz R. Matrix metalloproteinase inhibitor properties of tetracyclines: therapeutic potential in cardiovascular diseases. Pharmacol Res 2011; 64: 551-560. [https://doi.org/10.1016/j.phrs.2011.05.005](https://doi.org/10.1016/j.phrs.2011.05.005)
21. Parente JM, Castro MM. Matrix metalloproteinase in the cardiovascular remodeling of hypertension: current insights and therapeutic potential. Metalloproteinases in Medicine 2018; 5: 1-11. <https://doi.org/10.2147/MNM.S104793>
22. Brenes-Salazar JA. 2015. Minocycline: a bacteriostatic antibiotic with pleiotropic cardioprotective effects. Can J Physiol Pharmacol 93: 863-866. DOI: 10.1139/cjpp-2014-0493.
23. Liu J, Xiong W, Baca-Regen L, Nagase H, Baxter BT. Mechanism of inhibition of matrix metalloproteinase-2 expression by doxycycline in human aortic smooth muscle cells. J Vasc Surg 2003; 38: 1376-1383. <https://doi.org/10.1016/S0741-5214(03)01022-X>
24. Lindeman JH, Abdul-Hussien H, van Bockel JH, Wolterbeek R, Kleemann R. Clinical trial of doxycycline for matrix metalloproteinase-9 inhibition in pateints with an abdominal aneurysm. Doxycycline selectively depletes aortic wall neutrophils and cytotoxic T cells. Circulation 2009; 119: 2209-2216. DOI: 10.1161/CIRCULATIONAHA.108.806505
25. Mosorin M, Juvonen J, Biancari F, Satta J, Surcel HM, Leinonen M, Saikku P, Juvonen T. Use of doxycycline to decrease the growth rate of abdominal aortic aneurysms: a randomized, double-blind, placebo-controlled pilot study. J Vasc Surg 2001; 34: 606–610. DOI: [10.1067/mva.2001.117891](https://doi.org/10.1067/mva.2001.117891)
26. Hackmann AE, Rubin BG, Sanchez LA, Geraghty PA, Thompson RW, Curci JA. A randomized, placebo-controlled trial of doxycycline after endoluminal aneurysm repair. J Vasc Surg 2008; 48: 519–526. DOI: [10.1016/j.jvs.2008.03.064](https://doi.org/10.1016/j.jvs.2008.03.064)
27. Curci JA, Mao D, Bohner DG, Allen BT, Rubin BG, Reilly JM, Sicard GA, Thompson RW. Preoperative treatment with doxycycline reduces aortic wall expression and activation of matrix metalloproteinases in patients with abdominal aortic aneurysms. J Vasc Surg 2000; 31: 325-342. <https://doi.org/10.1016/S0741-5214(00)90163-0>
28. Singh B, Ghosh N, Saha D, Sarkar S, Bhattacharyya P, Chaudhury K. Effect of doxycyline in chronic obstructive pulmonary disease-An exploratory study. Pulm Pharmacol Ther 2019; 58, 101831. DOI: 10.1016/j.pupt.2019.101831
29. Prins HJ, Daniels JM, Lindeman JH, Lutter R, Boersma WG. Effects of doxycycline on local and systemic inflammation in stable COPD patients, a randomized clinical trial. Respir Med 2016; 110: 46-52. [https://doi.org/10.1016/j.rmed.2015.10.009](https://doi.org/10.1016/j.rmed.2015.10.009)
30. Sabir N, Hussain T, Mangi MH, Zhao D, Zhou X. 2019. Matrix metalloproteinases: Expression, regulation and role in the immunopathology of tuberculosis. Cell Proliferat 2019; 52: e12649. <https://doi.org/10.1111/cpr.12649>
31. Walker NF, Clark SO, Oni T, Andreu N, Tezera L, Singh S., Saraiva L, Pedersen B, Kelly DL, Tree JA, D’Armiento JM, Friedland JS, Elkington PT. Doxycycline and HIV infection suppress tuberculosis-induced matrix metalloproteinases. Am J Respir Crit Care Med 2012;185: 989-997. <https://doi.org/10.1164/rccm.201110-1769OC>
32. Xu X, Abdalla T, Bratcher PE, Jackson PL, Sabbatini G, Wells JM, Lou XY, Quinn R, Blalock JE, Clancy JP, Gaggar, A. 2017. Doxycycline improves clinical outcomes during cystic fibrosis exacerbations. Eur Respir J 2017; 49: 1601102. https://doi.org/10.1183/13993003.01102-2016
33. Xu DH, Zhu Z, Fang Y. The Effect of a Common Antibiotics Doxycycline on Non-Healing Chronic Wound. Curr Pharm Biotechnol 2017;18: 360-364. DOI: 10.2174/1389201018666170519095339.
34. Wilcox JR, Covington DS, Paez N. Doxycycline as a modulator of inflammation in chronic wounds. Wounds 2012; 24: 339-349. PMID: 25876218
35. Stechmiller J, Cowan L, Schultz G. The role of doxycycline as a matrix metalloproteinase inhibitor for the treatment of chronic wounds. Biol Res Nurs 2010; 11: 336-344. [https://doi.org/10.1177/1099800409346333](https://doi.org/10.1177%2F1099800409346333)
36. Menke MN, Menke NB, Boardman CH, Diegelmann RF. Biologic therapeutics and molecular profiling to optimize wound healing. Gynecol Oncol 2008; 111 (Suppl 2):S87–S91. DOI: 10.1016/j.ygyno.2008.07.052
37. Acharya MR, Venitz J, Figg WD, Sparreboom A. Chemically modified tetracyclines as inhibitors of matrix metalloproteinases. Drug Resist Updat 2004; 7: 195–208. [https://doi.org/10.1016/j.drup.2004.04.002](https://doi.org/10.1016/j.drup.2004.04.002)
38. Sabino F, auf dem Keller U. Matrix metalloproteinases in impaired wound healing. Metalloproteinases in Medicine 2015; 2: 1-8. <https://doi.org/10.2147/MNM.S68420>
39. Sadler GM, Wallace HJ, & Stacey MC. Oral doxycycline for the treatment of chronic leg ulceration. Arch Dermatol Res 2012; 304: 487-493. <http://dx.doi.org/10.1007/s00403-012-1211-y>.
40. Cariati A, Piromalli E, Cariati P. Effects of compression therapy and antibiotics on lymphatic flow and chronic venous leg ulceration. Arch Dermatol Res 2012; 304: 497-498. doi: 10.1007/s00403-012-1220-x
41. Zhang C, Gong W, Liu H, Guo Z, Ge S. Inhibition of matrix metalloproteinase-9 with low-dose doxycycline reduces acute lung injury induced by cardiopulmonary bypass. Int J Clin Exp Med 2014; 7: 4975-4982. PMID: [25663995](https://www.ncbi.nlm.nih.gov/pubmed/25663995)
42. Simonetti O, Cirioni O, Lucarini G, Orlando F, Ghiselli R, Silvestri C, Brescini M, Provinciali M, Guerrieri M, Di Primio R, Giacometti A, Offidani A. Tigecycline accelerates staphylococcal-infected burn wound healing through matrix metalloproteinase-9 modulation. J Antimicrob Chemother 2012; 67: 191–201. DOI:10.1093/jac/dkr440
43. Lee CZ, Yao JS, Huang Y, Zhai W, Liu W, Guglielmo BJ, Lin E, Yang GY, Young WL. Dose–response effect of tetracyclines on cerebral matrix metalloproteinase-9 after vascular endothelial growth factor hyperstimulation. J Cerebr Blood F Met 2006; 26: 1157-1164. [https://doi.org/10.1038/sj.jcbfm.9600268](https://doi.org/10.1038%2Fsj.jcbfm.9600268)
44. Bach DR, Tzovara A, Vunder J. Blocking human fear memory with the matrix metalloproteinase inhibitor doxycycline. Mol Psychiatry 2018; 23: 1584-1589. DOI: 10.1038/mp.2017.6
45. Siller SS, Broadie K. Matrix metalloproteinases and minocycline: Therapeutic avenues for fragile X syndrome. Neural Plasticity 2012, Article ID 124548. DOI: 10.1155/2012/124548
46. Kendre G, Raghavan R, Cheriyamundath S, Madassery J. Tetracycline and glutathione inhibit matrix metalloproteinase activity: an in vitro study using culture supernatants of L929 and dalton lymphoma cell lines. J Cancer Res 2013; Article ID 328134. [https://doi.org/10.1155/2013/328134](https://doi.org/10.1155/2013/328134)
47. Moir LM, Ng HY, Poniris MH, Santa T, Burgess JK. Oliver BGG, Krymskaya VP, Black JL Doxycycline inhibits matrix metalloproteinase‐2 secretion from TSC2‐null mouse embryonic fibroblasts and lymphangioleiomyomatosis cells. Br J Pharmacol 2011; 164: 83-92. DOI: [10.1111/j.1476-5381.2011.01344.x](https://doi.org/10.1111/j.1476-5381.2011.01344.x)
48. Samartzis EP, Fink D, Stucki M, Imesch P. Doxycycline reduces MMP-2 activity and inhibits invasion of 12Z epithelial endometriotic cells as well as MMP-2 and -9 activity in primary endometriotic stromal cells in vitro. Reprod Biol Endocrinol 2019; 17, 38. <https://doi.org/10.1186/s12958-019-0481-z>
49. Brown DL, Desai KK, Vakili BA, Nouneh C, Lee HM, Golub LM. Clinical and biochemical results of the metalloproteinase inhibition with subantimicrobial doses of doxycycline to prevent acute coronary syndromes (MIDAS) pilot trial. Arterioscler Thromb Vasc Biol 2004; 24: 733-738. DOI: [10.1161/01.str.0000038098.04291.f6](https://doi.org/10.1161/01.str.0000038098.04291.f6)
50. Axisa B, Loftus IM, Naylor AR, Goodall S, Jones L, Bell PR, Thompson MM. Prospective, randomized, double-blind trial investigating the effect of doxycycline on matrix metalloproteinase expression within atherosclerotic carotid plaques. Stroke 2002; 33: 2858-2864. DOI: [10.1161/01.str.0000038098.04291.f6](https://doi.org/10.1161/01.str.0000038098.04291.f6)
51. Lee HH, O'Malley MJ, Friel NA, Chu CR. Effects of doxycycline on mesenchymal stem cell chondrogenesis and cartilage repair. Osteoarthr Cartilage 2013; 21: 385-393. DOI: 10.1016/j.joca.2012.11.010
52. Franco GC, Kajiya M, Nakanishi T, Ohta K, Rosalen PL, Groppo FC, Ernst CWO, Boyesen JL, Bartlett JD, Stashenko P, Taubman MA , Kawai T. 2011. Inhibition of matrix metalloproteinase-9 activity by doxycycline ameliorates RANK ligand-induced osteoclast differentiation in vitro and in vivo. Exp Cell Res 2011; 317: 1454-1464. doi: [10.1016/j.yexcr.2011.03.014](https://dx.doi.org/10.1016%2Fj.yexcr.2011.03.014)
53. Brundula V, Rewcastle NB, Metz LM, Bernard CC, Yong VW. Targeting leukocyte MMPs and transmigration: minocycline as a potential therapy for multiple sclerosis. Brain 2002; 125: 1297-1308. <https://doi.org/10.1093/brain/awf133>
54. Sun T, Zhao N, Ni CS, Zhao XL, Zhang WZ, Su X, Zang DF, Gu Q, Sun BC. Doxycycline inhibits the adhesion and migration of melanoma cells by inhibiting the expression and phosphorylation of focal adhesion kinase (FAK). Cancer Lett 2009; 285: 141-150. doi: 10.1016/j.canlet.2009.05.004
55. Corps AN, Harrall RL, Curry VA, Hazleman BL, Riley GP. Contrasting effects of fluoroquinolone antibiotics on the expression of the collagenases, matrix metalloproteinases (MMP)-1 and-13, in human tendon-derived cells. Rheumatology 2005; 44: 1514-1517. doi:10.1093/rheumatology/kei087
56. Bujor AM, Haines P, Padilla C, Christmann RB, Junie M, Sampaio-Barros PD, Lafyatis R, Trojanowska, M. Ciprofloxacin has antifibrotic effects in scleroderma fibroblasts via downregulation of Dnmt1 and upregulation of Fli1. Int J Mo Med 2012; 30: 1473-1480. <https://doi.org/10.3892/ijmm.2012.1150>
57. Ruben EC, Manuel VR, Agustin OR, Huerta, M., Antonio FM, Ivan DE. Ciprofloxacin utility as antifibrotic in the skin of patients with scleroderma. J Dermatol 2010; 37: 323-329.

<https://doi.org/10.1111/j.1346-8138.2010.00826.x>

1. Reviglio VE, Hakim MA, Song JK, O'Brien TP. Effect of topical fluoroquinolones on the expression of matrix metalloproteinases in the cornea. BMC Ophthalmol 2003; 3: 10. DOI: [10.1186/1471-2415-3-10](https://doi.org/10.1186/1471-2415-3-10)
2. Sharma C, Velpandian , Baskar Singh S, Ranjan Biswas N, Bihari Vajpayee R, Ghose S. Effect of fluoroquinolones on the expression of matrix metalloproteinase in debrided cornea of rats. Toxicol Mech Methods 2011; 21: 6-12. DOI: 10.3109/15376516.2010.529183
3. Pfister W, Eick S. 2009. Moxifloxacin has a good antibacterial effect on pathogenic periodontal bacteria, but does not modulate the release of matrix metalloproteinase-3 in gingival fibroblasts. Infection 2009; 37 (Suppl 4): 62-63. ISSN 0300-8126
4. Roderfeld M, Rath T, Schulz R, Seeger W, Tschuschner A, Graf J, Roeb E. Serum matrix metalloproteinases in adult CF patients: Relation to pulmonary exacerbation. J Cyst Fibros 2009; 8: 338-347. [https://doi.org/10.1016/j.jcf.2009.06.001](https://doi.org/10.1016/j.jcf.2009.06.001)
5. Xu Y, WangL, Zimmerman MD, ChenK-Y, HuangL, Fu DJ, Kaya F, Rakhilin N, Nazarova EV, Bu P, Dartois V, Russell DG, Shen X. Matrix metalloproteinase inhibitors enhance the efficacy of frontline drugs against *Mycobacterium tuberculosis*. PLoS Pathog 2018; 14(4): e1006974. https://doi.org/10.1371/journal.ppat.1006974
6. RandL, Green JA, Saraiva L, Friedland JS, Elkington PT. Matrixmetalloproteinase-1 is regulated in tuberculosis by a p38 MAPK-dependent, p-aminosalicylic acid-sensitive signaling cascade. J Immunol 2009; 182: 5865–5872. http://dx.doi.org/10.4049/jimmunol.0801935.
7. Ambrosch A, Halevy D, Fwity B, Brin T, Lobmann R. Effect of daptomycin on local interleukin-6, matrix metalloproteinase-9, and metallopeptidase inhibitor 1 in patients with MRSA-infected diabetic foot. Int J Low Extrem Wounds 2013; 12: 100-105. [https://doi.org/10.1177/1534734613490506](https://doi.org/10.1177%2F1534734613490506)
8. Simonetti O, Lucarini G, Morroni G, Orlando F, Lazzarini R, Zizzi A, Brescini L, Provinciali M, Giacometti A, Offidani A, Cirioni O. Dalbavancin and wound healing: new evidences/insights in a mouse model of skin infection. Antimicrob Agents Chemother 2020; 64:e02062-19. https://doi.org/10.1128/AAC.02062-19.
9. Li S, Li C, Ryu HH, Lim SH, Jang WY, Jung S. Bacitracin inhibits the migration of U87-MG glioma cells via interferences of the integrin outside-in signaling pathway. J Korean Neurosurg Soc 2016; 59:106-116. http://dx.doi.org/10.3340/jkns.2016.59.2.106
10. Chan-on W, Huyen NTB, Songtawee N, Suwanjang W, Prachayasittikul S, Prachayasittikul V. Quinoline-based clioquinol and nitroxoline exhibit anticancer activity inducing FoxM1 inhibition in cholangiocarcinoma cells. Drug Des Devel Ther 2015; 9: 2033-2047. doi: [10.2147/DDDT.S79313](https://dx.doi.org/10.2147%2FDDDT.S79313)
11. Mitrovic A, Kos J. Nitroxoline: repurposing ist antimicrobial to antitumor application. Acta Biochimica Polonica 2019; 66: 521-532. https:// doi.org/10.18388/abp.2019_2904
